# Continuous Flow-Mode Synthesis of Aromatic Amines in a 3D-Printed Fixed Bed Reactor Loaded with Amino Sugar-Stabilized Re Apparent Nanoparticles

**DOI:** 10.3390/molecules30183782

**Published:** 2025-09-17

**Authors:** Patrick Niyirora, Joanna Wolska, Mateusz M. Marzec, Krystian Sokolowski, Anna Leśniewicz, Piotr Jamróz, Anna Dzimitrowicz, Andrzej Bernasik, Piotr Cyganowski

**Affiliations:** 1 Department of Process Engineering and Technology of Polymer and Carbon Materials, Wroclaw University of Science and Technology, 27 Wybrzeze St. Wyspianskiego, 50-370 Wroclaw, Poland; niyipa05@gmail.com (P.N.); joanna.wolska@pwr.edu.pl (J.W.); 2 Academic Centre for Materials and Nanotechnology, AGH University of Krakow, Mickiewicza Av. 30, 30-059 Kraków, Poland; marzecm@agh.edu.pl (M.M.M.); krysok@agh.edu.pl (K.S.); bernasik@agh.edu.pl (A.B.); 3 Department of Analytical Chemistry and Chemical Metallurgy, Wroclaw University of Science and Technology, 27 Wybrzeze St. Wyspianskiego, 50-370 Wroclaw, Poland; anna.lesniewicz@pwr.edu.pl (A.L.); piotr.jamroz@pwr.edu.pl (P.J.); anna.dzimitrowicz@pwr.edu.pl (A.D.); 4 Faculty of Physics and Applied Computer Science, AGH University of Krakow, Mickiewicza Av. 30, 30-059 Kraków, Poland

**Keywords:** nitroaromatics, reduction, rhenium, nanocatalyst, flow-mode processes

## Abstract

In industrial processes, catalysts—materials that speed up chemical reactions without being consumed—are essential. The goal of this research was to create two new rhenium-based nanocomposite catalysts that can effectively and sustainably reduce nitroaromatic compounds to aromatic amines in continuous-flow systems. Nitroaromatic hydrocarbons (NACs), widely used in manufacturing pharmaceuticals, insecticides, and herbicides, often contaminate soil and water, posing significant environmental and health risks. However, their reduction to aromatic amines enables potential industrial reuse. In this study, we synthesized two nanocomposite catalysts based on a copolymer functionalized with N-methyl-D-glucamine, embedded with rhenium (Re)-based apparent nanoparticles, and used them to reduce the NACs in continuous-flow mode to their aromatic amines using newly designed and stereolithographic (SLA) 3D-printed reactors. Advanced characterization techniques were employed to evaluate their structure, morphology, and catalytical performance. Catalyst 1, prepared from a self-modified Purolite D4869 resin and characterized by higher Re loading, exhibited superior conversion rates in batch mode (k_1_ up to 1.406 s^−1^). In contrast, Catalyst 2, based on a commercial NMDG-functionalized Dowex resin with a mesoporous structure, demonstrated remarkable stability and catalytic capacity under continuous flow (up to 1.383 mmol_NAC_ mL_cat_^−1^). Overall, Catalyst 1 was found to be better suited for rapid batch reactions, whereas Catalyst 2 was found to be more appropriate for long-term flow applications, offering a sustainable route for the efficient conversion of nitroaromatic compounds into valuable aromatic amines. The reactors enabled the efficient conversion of NACs into aromatic amines while enhancing process sustainability and efficiency.

## 1. Introduction

Catalytic hydrogenation is one of the most important industrial processes, enabling the synthesis of the fine chemical products necessary for a number of civilization’s major achievements [1,2,3]. In this context, aromatic amines (AAMs) are among the most important products in the hydrogenation process. These fine chemicals are necessary for the production of a variety of chemicals, such as large-scale pharmaceuticals (e.g., paracetamol, ibuprofen), and a variety of antibiotics (e.g., linezolid, chloramphenicol) [4,5]. AAMs are usually obtained as a result of the reduction of nitroaromatic compounds (NACs), which should preferably be carried out in the presence of a catalyst [6].

In recent years, numerous homogenous and heterogeneous catalysts based on metallic nanoparticles have been recognized as valid tools for achieving the direct reduction of –NO_2_ to –NH_2_ groups in batch- and continuous-mode processes [7,8,9]. Although economically feasible, batch-mode processes have limited scalability and efficiency, which frequently present difficulties in maintaining reaction control. On the other hand, continuous-flow systems are especially well-suited for industrial applications due to their many benefits, including increased safety, improved scalability, and improved control over reaction parameters [10,11,12,13,14,15,16,17]. These latter examples, when considered for catalytic hydrogenation processes, require a suitable heterogeneous catalyst.

In this context, recent research has investigated embedding nanoparticles into support materials, improving the stability and reusability of the catalyst. Metal–organic frameworks, polymers, and carbon-based materials are examples of supports that enhance mass transfer, maintain structural stability, and prevent catalyst deactivation [18,19,20,21,22,23]. Rhenium-based heterogeneous catalysts have shown promise in the hydrogenation of NACs, with sub-nanostructures offering improved selectivity and efficiency [24,25]. These may enable the carrying out of a continuous flow-mode hydrogenation process aimed at the production of AAMs from NACs.

However, the efficient reduction of NACs in continuous-flow mode is still technically difficult, as efficient and selective catalytic systems must be able to endure extended exposure to reaction conditions without experiencing appreciable leaching or the deactivation of the active catalyst material. In this context, this work explores a new catalytic system based on an N-methyl-D-glucamine (NMDG) functionalized catalyst loaded with rhenium (Re) active sites. Although rhenium-based nanocatalysts have shown activity in nitroaromatic reductions, there are no reports of their stabilization in amino sugar-functionalized supports for continuous-flow applications. Addressing this gap, we explore the use of NMDG functionalities to immobilize rhenium oxyanions and promote the formation of sub-nanometer Re clusters. This unique stabilization strategy is expected to create abundant nucleation sites, suppress agglomeration, and enhance catalyst durability. By embedding these catalysts into 3D-printed tubular reactors, we aim to establish a robust continuous-flow system for the sustainable hydrogenation of nitroaromatic compounds into aromatic amines.

The synthetic protocol aimed at the synthesis of the new heterogeneous catalyst involved the application of commercial NMDG-functionalized DOWEX XUS43594 chelating resin. The resin was then loaded with ReO_4_^−^ oxyanion, which was subsequently reduced via NMDG functionalities to Re apparent nanoparticles, the catalytically active sites for the hydrogenation processes. Here, the term “apparent nanoparticles” is introduced to describe sub-nanometer rhenium clusters that occasionally aggregate into ultra-small structures. Due to their unique morphology and the absence of clear terminology for materials of this scale, these clusters manifest as highly dispersed atomic assemblages that resist agglomeration, offering abundant active sites and thereby improving catalytic reactivity. The application of rhenium (Re) apparent nanoparticles provides a strong catalytic system for NAC reduction when embedded in a styrene–divinylbenzene copolymer. While the polymer support provides mechanical stability and promotes effective mass transfer, rhenium apparent nanoparticles were chosen for their catalytic activity and capacity to tolerate the conditions necessary for successful NAC reduction [24,25,26,27,28].

The resin with NMDG functionalities was selected due to its multiple oxygen and nitrogen donor atoms, which enable the effective chelation of oxyanions, their stabilization, and the subsequent reduction to the corresponding apparent nanoparticles [29,30,31,32,33]. NMDG has previously been investigated for its ability to selectively bind oxyanions such as arsenate, utilizing its protonated amine and hydroxyl groups in complexation mechanisms [29]. Additionally, similar functionalities in poly-L-histidine resins have demonstrated effective metal chelation, underscoring the importance of donor atoms in coordinating metal species [30]. However, its application in reducing rhenium oxyanions (ReO_4_^−^) and stabilizing sub-nanometric rhenium structures has not yet been reported in the literature. Hence, it is hypothesized that this novel approach may leverage the synergy between NMDG functional groups and rhenium apparent nanoparticles, significantly increasing the number of nucleation sites. This prevents nanomaterial agglomeration, enhances chemical stability by limiting oxygen exposure, and ultimately improves catalytic performance. The resulting heterogeneous catalysts demonstrate high NAC hydrogenation activity, providing a scalable and cost-effective alternative to conventional gold- and platinum-based catalysts.

## 2. Results and Discussion

### 2.1. Characterization of the Synthesized Catalytic Materials

#### 2.1.1. FT-IR Analysis

**Figure 1:**
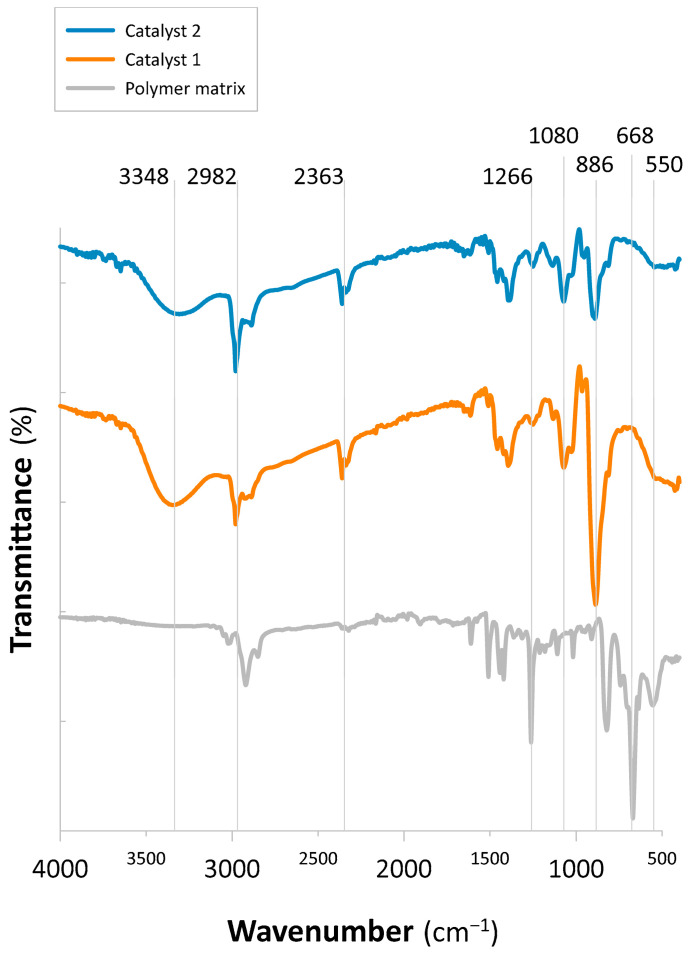
FT-IR graph for S-co-DVB Purolite D4869 and NMDG-modified polymers 1 and 2.

Fourier transformation infrared (FT-IR) spectra (Figure 1, Table S2) were used to verify the presence of NMDG functionalities in the commercial resin (Catalyst 2) and to assess their incorporation into the self-modified polymer (Catalyst 1). For comparison, the unmodified polymeric matrix used for catalyst synthesis of Catalyst 1 was also analyzed. Characteristic bands at 554 cm^−1^ and 671 cm^−1^, initially observed in the chloromethylated S-co-DVB copolymer, disappeared after modification and were likewise absent in the commercial XUS43594 resin. These spectral changes indicate that the –CH_2_Cl groups originally present in the matrices were successfully transformed during the functionalization process [34].

Further, C=C bending was noted in the NMDG-functionalized Catalyst 1 and Catalyst at 822 cm^−1^, 888 cm^−1^, and 890 cm^−1^ [34], while O=C=O stretching bands were observed at 2363 cm^−1^ and 2360 cm^−1^, respectively. Amine groups were present because the C– stretching bands shifted to 2979 cm^−1^, and new N–H and O–H stretching peaks appeared at 3367 cm^−1^, 3365 cm^−1^, and 3349 cm^−1^ [34]. Furthermore, in the modified resins, the O–H stretching band at 1073 cm^−1^ demonstrated the presence of polyol moieties from NMDG [34,35].

For both Catalyst 1 and Catalyst 2, the FT-IR spectra showed characteristic N–H and O–H stretching vibrations together with O–H bending signals, confirming the presence of amine groups and polyol moieties derived from NMDG. These features verify the successful modification of the polymer matrices and incorporation of NMDG functionalities.

#### 2.1.2. Electron Microscopy

**Figure 2:**
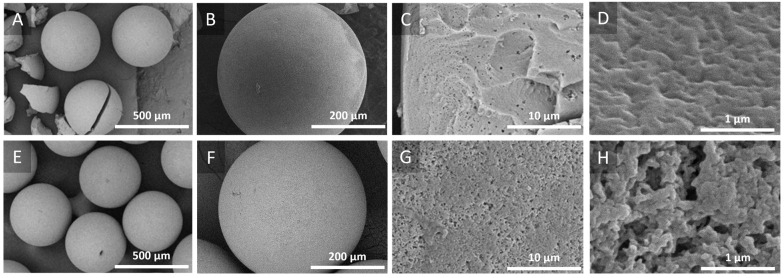
SEM images of (**A**–**D**) Catalyst 1 and (**E**–**H**) Catalyst 2.

The morphology and topography of the synthesized catalysts were estimated by scanning electron microscopy (SEM). As can be seen in Figure 2, a spherical morphology with suspected porous structures was revealed. Since the spherical shape guarantees appropriate flow characteristics (i.e., reduced hydrodynamic pressures), it is especially crucial for the flow-mode process being developed in this study. The fact that there are visible pores further demonstrates that the pore structure, which is essential for increasing the surface area, was not blocked by the modification protocol applied to this study or implemented by the Dowex resin manufacturer (Dow Chemical Co., Stade, Germany).

In this context, the surface area and porosity of the polymer-supported catalysts were determined using low-temperature nitrogen adsorption–desorption isotherms at 77 K. Based on this, Catalyst 1 showed very little porosity, with a BET surface area of 0.30 m^2^ g^−1^ and a total pore volume of 0.000145 cm^3^ g^−1^. In contrast, Catalyst 2 showed a BET surface area of 15.96 m^2^ g^−1^ and a total pore volume of 0.23 cm^3^ g^−1^. Additionally, mesoporous features with pore widths ranging from 1.99 nm to 50.21 nm were observed. The elemental compositions of Catalyst 1 and Catalyst 2, examined by EDX (Figure S6) and confirmed by ICP-OES, indicate that Catalyst 1 demonstrates a higher Re loading (275.8 mg g^−1^; 36.89 wt%) than Catalyst 2 (107.9 mg g^−1^; 26.65 wt%). Further, based on the EDX spectra (Figure S6), Catalyst 2 contained slightly more N (5.97 wt% vs. 4.53 wt%) and C (34.84 wt% vs. 32.32 wt%), indicating a more organic-rich matrix. These compositional differences suggest possible differences in surface interactions and catalytic activity.

**Figure 3:**
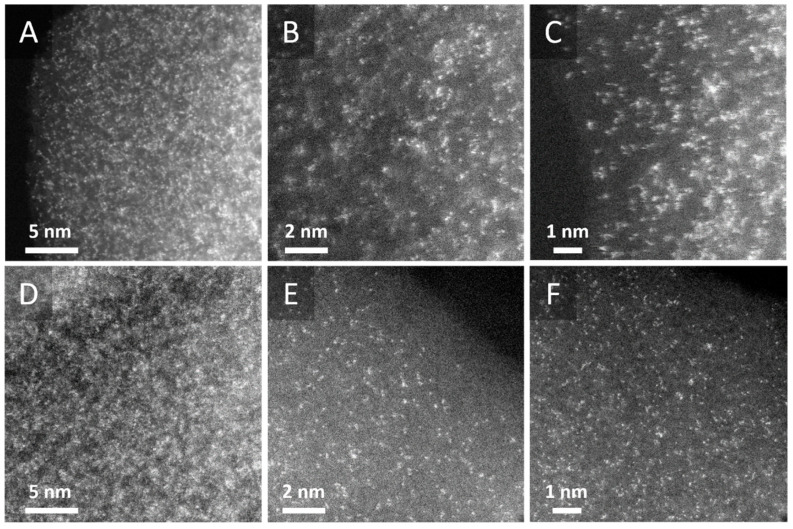
HRTEM/HAADF images of (**A**–**C**) Catalyst 1 and (**D**–**F**) Catalyst 2.

High-resolution transmission electron microscopy (HRTEM) was used to observe Re clusters. It was anticipated from earlier studies that Re would occasionally resemble apparent nanoparticles and form extremely small structures made up of atomic clusters [36]. Consequently, Re nanoparticles and Re apparent nanoparticles in the cross-sections of the polymers were observed using HRTEM assisted by a high-angle annular dark field detector (HAAFD). As seen in Figure 3, the images reveal clearly defined phase contrast, with bright spots indicating well-dispersed Re atom clusters that were occasionally grouped into very small Re apparent nanoparticles. This morphology facilitates the efficient distribution and integration of Re within the catalysts; hence, it is expected that they may demonstrate an improved catalytic performance.

#### 2.1.3. Phase Composition

The X-ray powder diffraction (XRD) diffractograms of both catalysts (Figure S7) showed a broad peak centered around 20° 2θ, which is characteristic of predominantly amorphous polymer matrices. The peak intensity (higher for Catalyst 1 than for Catalyst 2) indicates the presence of small, disordered crystalline domains and varying degrees of short-range order within the amorphous structure.

**Figure 4:**
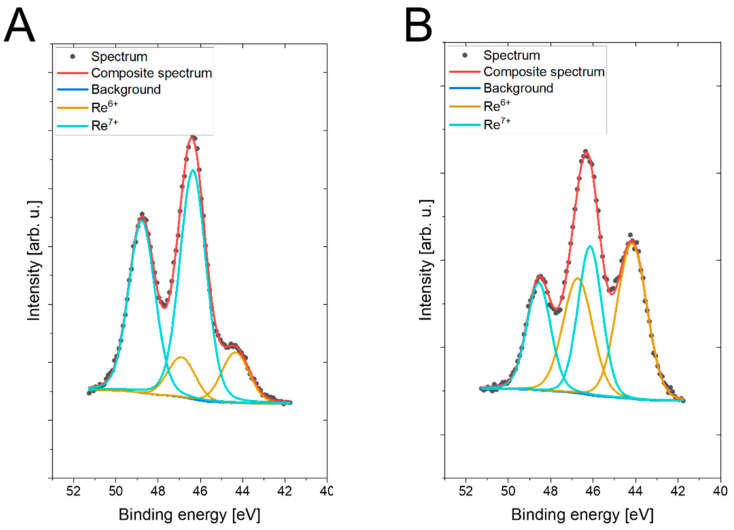
Re 4f XPS deconvoluted spectra for (**A**) Catalyst 1 and (**B**) Catalyst 2.

X-ray photoelectron spectroscopy (XPS) analysis (Figure 4 and Figures S14–S16, Table 1) confirmed the surface composition and chemical environment of the catalysts. The C 1s, O 1s, N 1s, Cl 2p, and Si 2p spectra (Figures S14–S16) showed the expected signals corresponding to the polymer backbone and NMDG functionalities [37,38,39,40,41]. Importantly, the Re 4f spectra (Figure 4) revealed the presence of Re in +6 (ReO_3_) and +7 (Re_2_O_7_) oxidation states, while the detection of C–NH bonds verified the successful reaction of NMDG with the polymer matrices. Together, these findings confirm that rhenium species were effectively immobilized on the functionalized supports through oxyanion adsorption–reduction processes, resulting in highly dispersed catalytic sites.

To enable comparison between different techniques (XPS, ICP-OES, EDX), atomic percentages derived from XPS analysis were recalculated to weight percentages using Equation (S1) (please see Supplementary Information for details). Although it must be noted that both XPS and EDX results relate to surface loading, the values re-calculated (Table S4) in this way confirm consistency, validating that Catalyst 1 contained slightly higher Re loading than Catalyst 2.

Lastly, it must also be noted that the values of the water regained for both polymeric matrices used for the catalysts’ synthesis were estimated as 0.367 and 0.832 g_H2O_ g_polymer_^−1^ for the matrices of Catalyst 1 and Catalyst 2, respectively (Table S3). The ability of Catalyst 2’s matrix to capture more water will probably influence its catalytic performance by affecting the diffusion of reactants and the overall interaction between the catalysts and the reaction medium.

### 2.2. Determination of Catalytic Activity in Batch Mode

To evaluate the catalytic activity of the prepared catalysts, the reduction in 4-nitrophenol (4-NP) to 4-aminophenol (4-AP) was conducted as the model reaction. This is because this reaction only occurs in the presence of a catalyst, yielding 4-AP as the sole product. The reduction involves two main steps: the dissociation of sodium borohydride (NaBH_4_) into BH_4_^−^ ions, followed by the reduction of 4-NP to 4-AP, catalyzed by the catalyst [42,43,44]. UV–Vis spectral analysis (Figures S8 and S9) showed a gradual decrease in absorbance at 400 nm, accompanied by the appearance of a new peak at 295 nm, corresponding to 4-aminophenol, confirming the reduction of –NO_2_ groups to –NH_2_ groups. Similar behavior was observed for other nitroaromatic substrates, i.e., 2,4-dinitrophenol (2,4-DNP), 2,4,6-trinitrophenol (2,4,6-TNP), 2-nitroaniline (2-NA), 4-nitroaniline (4-NA), and nitrobenzene (NB) (Table 2), at their characteristic wavelengths (Figures S8 and S9). Notably, the reaction profiles differed between the two catalysts: reductions over Catalyst 1 (Figure S8) proceeded in a single step, whereas reductions over Catalyst 2 (Figure S9) exhibited an induction period, likely due to diffusion limitations within its mesoporous structure.

**Figure 5:**
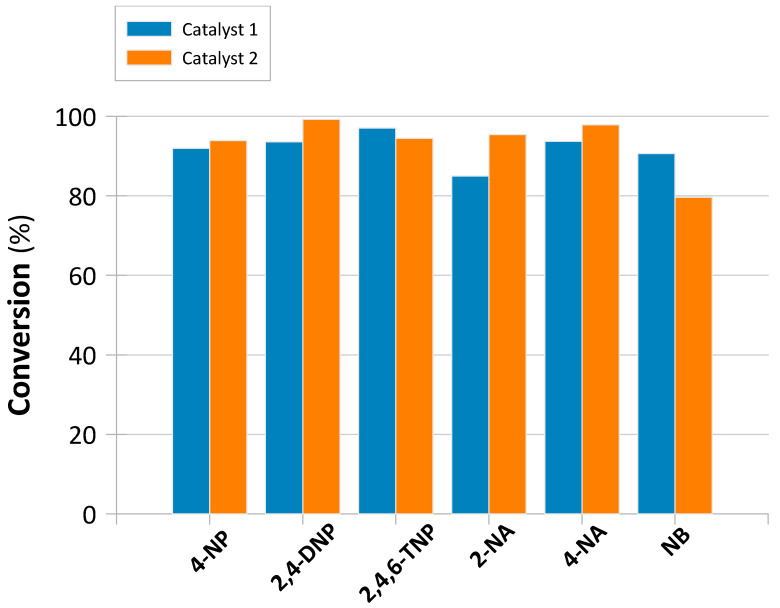
Comparison of NAC conversions (%) in batch processes carried out over Catalyst 1 and Catalyst 2.

The kinetic parameters are summarized in Table 2 and Figure 5. Further, the kinetic plots used to derive rate constants (*k*_1_) are provided in Figures S8 and S9. Based on these data, Catalyst 1 showed higher reaction rate constants (*k*_1_ = 0.263–1.406 s^−1^) and consistently greater NAC conversion compared to Catalyst 2 (*k*_1_ = 0.040–0.261 s^−1^). TOF analysis further confirmed the higher molar activity of Catalyst 1 in batch mode as well. Both the smaller catalytic activity and the induction periods of the reactions carried out over Catalyst 2 again indicate possible diffusion limitations caused by Catalyst 2’s mesoporous characteristics. This obvious drawback in the batch-mode system is expected, however, to be an advantage in the flow-mode system, as Catalyst 2 may reveal enhanced stability and capacity.

### 2.3. Continuous Flow-Mode Reduction of NACs

**Figure 6:**
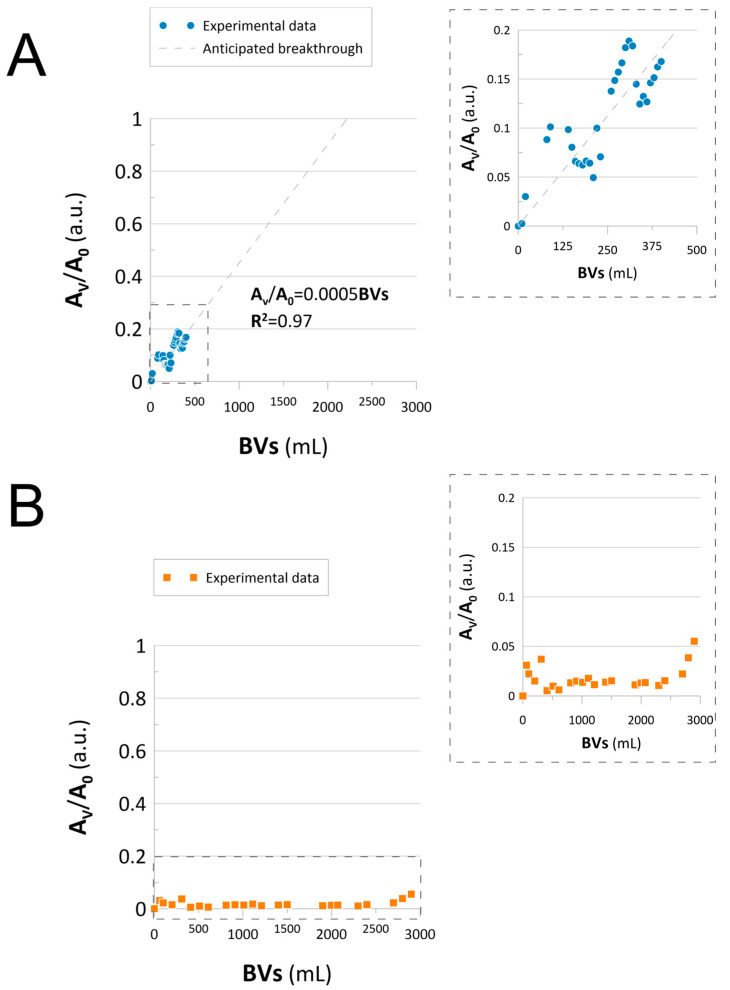
Breakthrough curves for (**A**) Catalyst 1 and (**B**) Catalyst 2 in the flow-mode reduction of 4-NP. Reaction conditions: C_4-NP_ = 0.1 mmol L^−1^; C_NaBH4_ = 0.26 mol L^−1^ (4-NP:NaBH_4_ = 500:1); flow rate = 1 mL min^−1^.

Because Catalyst 1 and Catalyst 2 turned out to be well-suited for the hydrogenation of NACs, they were applied in the continuous flow-mode process. First, both catalysts were used in an experimental setup, in which a 3D-printed reactor loaded with a catalytic bed was fed in a counter-flow with the mixture of 4-NP and NaBH_4_ solution. Catalytic reduction under flow conditions was monitored in real time by collecting the reactor effluents, which were further subjected to UV–Vis analysis. The collected spectra are displayed in the Supplementary Materials (Figures S11–S13). These were then used to draw the reactors’ breakthrough curves displayed in Figure 6.

Due to the substantial amounts of processed solutions, the test was carried out until reactor breakthrough could be clearly observed, but no more than 3000 mL of the reagents was processed. Flow-mode experiments revealed clear differences between the two catalysts. Catalyst 1 began losing capacity from the start of the operation (Figure 6A), with a 20% breakthrough observed at 400 mL bed volumes (BVs) and complete exhaustion projected at 2600 mL, corresponding to a capacity of 0.343 mmol_NAC_ mL_cat_^−1^ (Figure 6A). In contrast, Catalyst 2 showed no breakthrough, even after processing 3000 mL of the 4-NP solution (Figure 6B). These results indicate that Catalyst 2 maintains catalytic activity over the extended operation and is more efficient for high-volume continuous processing.

At this point, it must be noticed that despite Catalyst 1 performing better in batch mode, in the present scenario, Catalyst 2 is a much better fit. The reason for this may be linked to the capacity offered by the mesoporous structure of Catalyst 2’s polymeric matrix. In this context, despite an induction period shown in the batch mode process, in the flow-mode system, Catalyst 2 revealed enhanced catalytic capacity, indicating its ability to handle higher volumes of NACs. It is worth pointing out that the column effluents reveal an absorbance at ~295 nm (Figures S11 and S12), attributed to the 4-NP reduction product, namely, 4-aminophenol. This, linked to the lack of column breakthrough in the case of Catalyst 2, could recommend this process as a unique approach to the production of pure aromatic amines from NACs.

#### Catalysts Performance and Stability

The contrasting performance of Catalyst 1 and Catalyst 2 in batch-versus continuous-flow mode highlights the importance of catalyst structure and morphology for reactor design. While Catalyst 1, with its higher rhenium loading, achieved faster reaction rates and higher conversions in batch experiments, its catalytic capacity decreased steadily under flow conditions, likely due to diffusion limitations and gradual surface deactivation. By contrast, Catalyst 2 exhibited slower kinetics in batch mode but maintained remarkable stability and capacity under continuous operation. This can be attributed to its mesoporous polymeric matrix, which facilitates mass transport, prevents rapid leaching of active sites, and ensures sustained reactivity over extended volumes of processed solution. These findings demonstrate that Catalyst 1 is best suited for rapid, small-scale reductions, whereas Catalyst 2 is more advantageous in continuous flow applications where stability and throughput are critical. Together, the results emphasize the need to match catalyst design not only to intrinsic activity but also to the intended reactor configuration.

At this point, it should also be noted that the column flow-mode process provided the breakthrough curves, which are functionally equivalent to reusability testing under continuous conditions. These results clearly distinguished the two systems: Catalyst 1 showed gradual deactivation and limited capacity, while Catalyst 2 maintained stable performance over extended reagent volumes. We therefore consider the flow-mode breakthrough analysis to be the most relevant and realistic demonstration of catalyst durability in the context of this work.

**Figure 7:**
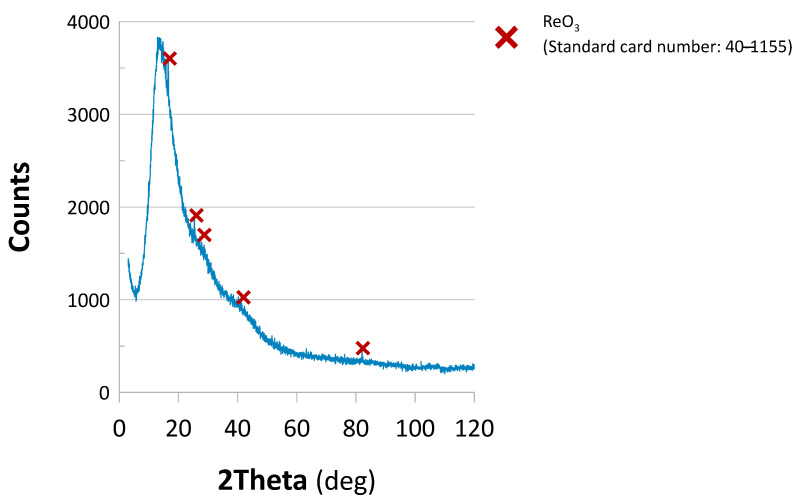
XRD diffractogram of Catalyst 2 taken out of the reactor using the flow-mode process of 4-NP reduction.

To verify the impact of the reaction environment on the catalysts’ phase composition. Catalyst 2 underwent XRD analysis for the sample taken out of the reactor used in the flow-mode process. Based on the spectrum displayed in Figure 7, there is a series of noticeable peaks attributed to the presence of ReO_3_ (standard reference card number: 40-1155). Because the unused Catalyst 2 (Figure S7) revealed an amorphous structure, these peaks suggested that rhenium oxide species developed during the reduction reaction. Nevertheless, as displayed in Figure 6, at the point of taking the measurement, Catalyst 2 was still operational.

### 2.4. Flow-Mode Catalytic Reduction in Multicomponent System

Because of Catalyst 2’s performance in the flow-mode process of 4-NP reduction (Figure 6B), as well as its ability to convert other NACs (Figures S9 and S10), the catalyst was also employed in the continuous process aimed at the catalytic reduction of the NACs mixture. In this process, a feed solution containing 4-NP, 2,4-DNP, 2,4,6-TNP, 2-NA, 4-NA, and NB was passed through the reactor, and the effluents were collected and analyzed using UV–Vis.

**Figure 8:**
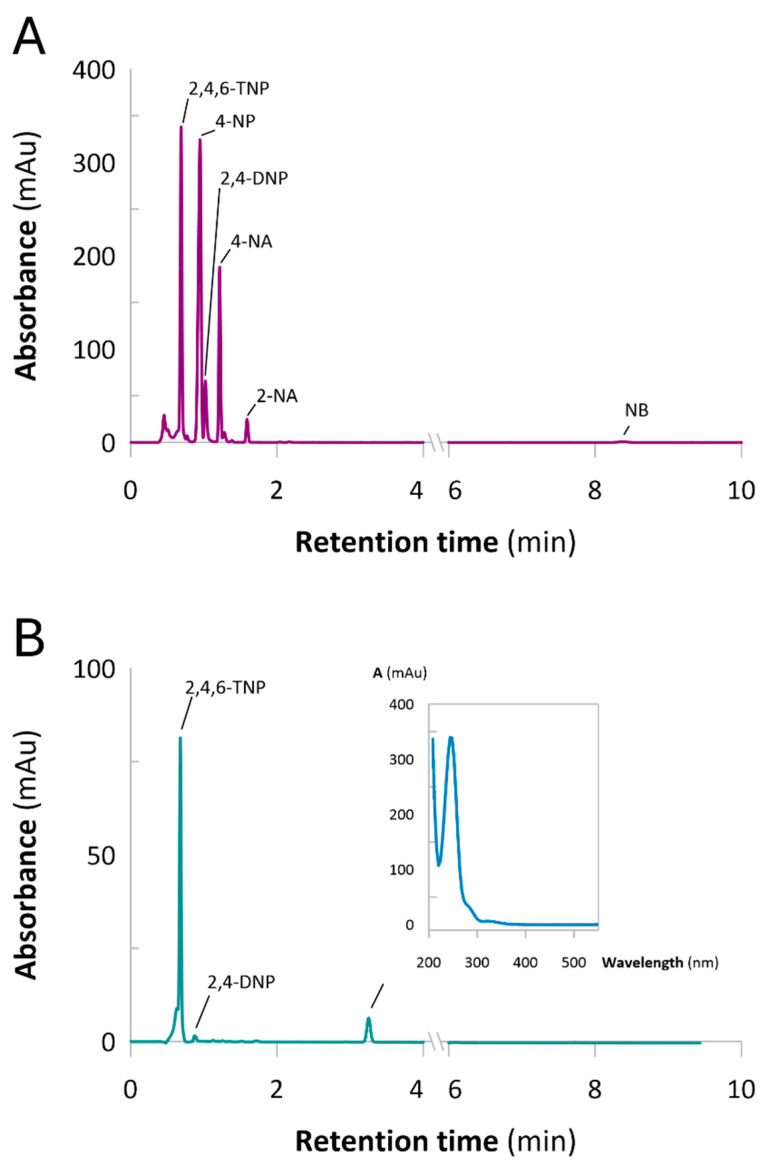
HPLC-DAD chromatograms of (**A**) NACs mixture fed into the column and (**B**) column effluent collected at BVs = 10 mL. The column contained a Catalyst 2 bed. Retention times: 2,4,6-TNP—0.690 min; 4-NP—0.947 min; 2,4-DNP—1.020 min; 4-NA—1.217 min; 2-NA—1.590 min; NB—8.380.

The UV–Vis spectra of the column effluents (Figure S13) showed absorbance in the 290–300 nm range, consistent with amino group-containing products, together with a minor signal near 400 nm, indicating the early leakage of residual nitro compounds. To better assess Catalyst 2’s performance, the effluents were analyzed by HPLC-DAD. The chromatograms (Figure 8) revealed that from the start of operation, only 2,4,6-TNP and 2,4-DNP appeared in the outflow, while a new component with a retention time of 3.25 min was detected, corresponding to an aromatic amine. Across the entire tested range (10–1100 mL), Catalyst 2 efficiently reduced 4-NP, 4-NA, 2-NA, and NB but showed poor activity toward 2,4,6-TNP and incomplete conversion of 2,4-DNP. This selectivity is likely related to the strong electron-withdrawing effects of multiple nitro groups in these substrates, which hinder electron transfer during the reduction process [42,43,44].

## 3. Materials and Methods

### 3.1. Reagents and Materials

Two N-methyl-D-glucamine (NMDG) functionalized chelating resins (Figure S1) were used in this study as support for Re-based catalysts. The first one was synthesized by modifying chloromethylated styrene-co-divinylbenzene (S-co-DVB) copolymer Purolite D4869 with NMDG (99.0%, Sigma-Aldrich, Fluka Biochemika 66930, St. Louis, MO, USA). The second one was a commercial NMDG-functionalized DOWEX XUS43594 chelating resin (Dow Chemical Co., Stade, Germany).

All reagents were of analytical grade or higher. Solvents, reducing agents, and precursors, including 1,4-dioxane, sodium borohydride, and ammonium perrhenate, were obtained from Sigma-Aldrich (Merck, Warsaw, Poland) and used as received. Additional solvents and purification reagents, such as water, toluene, acetone, ethanol, and reverse osmosis (RO) water, were sourced from Avantor Performance Materials (Gliwice, Poland). All of the nitroaromatic compounds named in this study (analytical grade) were also acquired from Sigma-Aldrich (Merck, Poland).

### 3.2. Analytical Methods

Nanocomposite catalysts were evaluated using several instrumental techniques. Fourier transform infrared spectroscopy (FT-IR) was performed on a Jasco FT-IR 4700 (Hachioji, Japan) with a spectral range of 4000 to 400 cm^−1^. High-resolution transmission electron microscopy (HR-TEM) was conducted using an FEI TITAN^3^ (Thermo Fisher company, Waltham, MA, USA) microscope. X-ray photoelectron spectroscopy (XPS) analysis was carried out on a PHI VersaProbe II system (Physical Electronics, Chanhassen, MN, USA) with monochromatic Al Kα (1486.6 eV) X-rays. Survey scans and high-resolution spectra for the C 1s, O 1s, Re 4f, Si 2p, and Cl 2p regions were obtained, with charge compensation via Ar^+^ ions and electrons. Elemental analysis was conducted using a Vario Elementar Analysensysteme GmbH instrument (Langenselbold, Germany). For the analysis of the porous structures of synthesized catalysts, low-temperature nitrogen adsorption–desorption isotherms were analyzed. These were recorded using a Micromeritics ASAP 2020 Plus instrument (Micromeritics Instrument Corporation, Norcross, GA, USA). Catalytic activity was modeled using UV–visible spectrophotometry on a Jasco V530 (Frederick, MD, USA) spectrometer. A 3D stereolithographic (SLA) printer Formlabs Form 3 was used for catalytic-separation system development. The flow-mode catalytic reaction was carried out with the aid of a Unipan peristaltic pump (Unipan, Warsaw, Poland).

The concentrations of the NACs in the mixtures were estimated using high-performance liquid chromatography (HPLC). This was conducted with the aid of a ThermoFisher (Frederick, MD, USA) Vanquish instrument equipped with a 4-channel gradient pump, an autosampler, and a diode array UV–Vis detector (DAD). The HPLC-DAD analysis was performed using 100 µL sample injection and gradient elution, with water and acetonitrile as components A and C, respectively. As the ion pairing eluent (component D), 0.05 mol L^−1^ formic acid–ammonium formate buffer (pH 4) was used. The elution was carried out over a C18-PFP column (Hypersil Gold, ThermoScientific, Franklin, MA, USA). The applied water–acetonitrile gradient ranged from 40:60 to 20:80, with a constant flow rate of 1 mL min^−1^. Chromatographic separation was performed for 10 min using UV–Vis detection at 275 nm.

### 3.3. Synthesis of Polymeric Materials Containing Rhenium Apparent Nanoparticles

Scheme S1 displays the detailed research protocol applied in this study. Two Re-based catalysts were prepared as follows (the steps of the procedure are displayed in Scheme S1, Figures S1 and S2). Catalyst 1 was synthesized in the following way: Chloromethylated S-co-DVB copolymer Purolite D4869 (5 g) was placed in a round-bottom flask, and a 1:1 H_2_O–1,4-dioxane mixture (50 mL) was added. The copolymer was swollen in this mixture for 24 h. Afterward, the flask and its contents were connected to a reflux condenser and heated up to 80 °C. Then, NMDG was added in an amount ensuring 5:1 molar excess of NMDG (0.190 mol or 37.10 g) compared to Cl in the Purolite D4869 (5.9 g wet resin contains 4.8 g of dry resin and 0.0380 mol of Cl). The so-prepared reaction was carried out at 80 °C for 24 h. After this, the NMDG-functionalized resin was filtrated and then extracted in H_2_O and then in 0.1 mol L^−1^ and 0.001 mol L^−1^ HCl, respectively. Then, swollen resin was contacted with 1.4406 g L^−1^ NH_4_ReO_4_ solution (1000 mg Re L^−1^) for 48 h and then filtrated and put in H_2_O for the next 48 h.

Catalyst 2 was synthesized in the following way: A commercial Dowex XUS43594 resin was swollen in 0.1 and 0.001 mol L^−1^ HCl and then introduced into 1.44 g L^−1^ NH_4_ReO_4_ solution (1000 mg Re L^−1^) for 48 h. Afterward, the Re-loaded resin was filtrated and put in H_2_O for the next 48 h.

The last steps of the above-described procedures enabled the reduction-coupled adsorption of ReO_4_^−^, which resulted in the reduction and precipitation of Re active sites in the polymeric matrix. Then, the samples were subjected to further procedures.

### 3.4. Catalytic Reduction of NACs in Batch Mode

The catalytic reduction of nitroaromatic compounds (NACs) was evaluated in batch experiments using UV–Vis spectrophotometry (Figure S3). A cuvette containing 2.7 mL of 0.1 mmol L^−1^ of 4-nitrophenol (4-NP) was used to record the absorbance peak at 318 nm. Following the addition of 0.5 mL of 0.26 mol L^−1^ NaBH_4_, a color shift assigned to the 4-nitrophenolate anion was recorded at 400 nm. Then, 0.05 g of Catalyst 1 or Catalyst 2 was added to enable the reduction of 4-NP. This reaction was observed by monitoring the gradual decrease in the band at 400 nm and the simultaneous appearance of a band at 295 nm assigned to the reaction product, 4-aminophenol (4-AP). The same procedure was applied by monitoring the bands at 275, 360, 380, and 410 nm for the reduction of nitrobenzene (NB), 2,4-dinitrophenol (2,4,-DNP), 2,4,6-trinitrophenol (2,4,6-TNP), 2-nitroaniline (2-NA), 4-nitroaniline (4-NA), and a mixed solution of all of the mentioned NACs (0. 16 mmol L^−1^ per component).

A large excess of NaBH_4_ was deliberately used to ensure pseudo-first-order kinetics, since without such an excess, the reaction rate would depend on both the NaBH_4_ and NAC concentrations. Under these conditions, the reaction could be modeled as a first-order reaction with the assumption that recorded absorbance values are directly proportional to NAC concentrations Equation (1):

(1) $$-\ln\frac{A_{t}}{A_{0}}=k_{1}t$$

where *k*_1_ is the rate constant of the reaction, *A*_0_ is the initial absorbance of 4-nitrophenolate anion, and *A_t_* is absorbance recorded at time *t*.

Conversion was calculated according to Equation (2), where *A_0_* is the initial absorbance of the nitroaromatic compound and *A_t_* is its absorbance at time t. In addition to kinetic modeling, catalytic activity was also evaluated in terms of turnover frequency (*TOF*), expressing the catalyst molar activity. This value was calculated according to Equation (3):

*Conversion* (%) = ((*A*_0_ − *A_t_*)/*A*_0_) × 100 (2)

*TOF* (h^−1^) = (*moles of substrate converted*)/(*moles of Re* × *reaction time*) (3)

Here, the moles of rhenium were determined from the Re content measured by Energy-dispersive X-ray (EDX) elemental analysis, and t corresponds to the reaction time (h). This calculation assumes that all Re atoms are catalytically active.

### 3.5. Stereolithographic (SLA) 3D Printing of Reaction Columns

**Figure 9:**
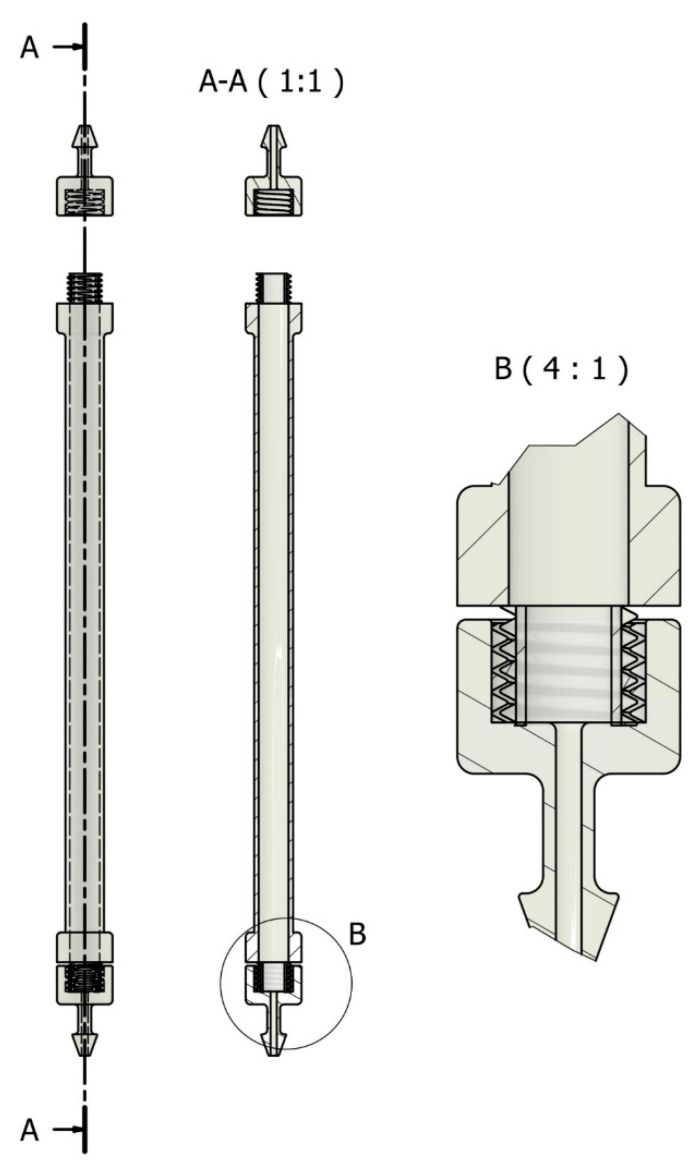
Design of the 3D-printed reactor applied for the continuous flow-mode process.

The reactor vessels were produced using stereolithographic (SLA) 3D printing technology. The process consisted of three primary steps: design, printing, and post-processing. Autodesk Inventor Professional 2024 was used to create the column design, which was then exported as an STL file and prepared for layer-by-layer printing using Formlabs PreForm 3.3 software (Figure S4). During the printing process, the SLA printer selectively exposed the liquid resin to UV light to cure each layer and create an accurate model. After printing, any remaining resin was removed from the columns using isopropyl alcohol in a FormWash cleaner, and it was post-cured in a FormCure oven to finally crosslink the resin (Figure S5) [45]. The final column used as the vessel for the catalytic reactor is displayed in Figure 9.

### 3.6. Catalytic Reduction of NACs in Continuous-Flow Mode

Each of the 3D-printed reactor vessels was loaded with Catalyst 1 or Catalyst 2 and then connected to a flow system with an adjusted flow rate of 1.25 mL min^−1^ (Figure S3 and Table S1). For the test, the columns were fed with 27 mL of NAC (5 mL NaBH_4_ mixed with 0.1 mmol L^−1^ NAC solution and 0.26 mol L^−1^ NaBH_4_) at the above-mentioned flow rate. The reaction progress was tracked by collecting effluent samples for UV–Vis spectrophotometric analysis at predetermined intervals of 10 mL passed through a column. For the test carried out for the NAC mixture, HPLC analysis of the reactor’s effluent was performed.

Breakthrough curves allowing for the estimation of the “catalytical capacity” of a reactor were constructed by plotting the ratio between the absorbance of an analyte in the column effluent collected at a passed volume (*A_v_*) and the absorbance in the feed solution (*A*_0_). In this case, the *A_v_/A*_0_ ratio was assumed to be equivalent to the concentration ratio as well. *A_v_/A*_0_ was then plotted against the function of catalyst bed volumes (*BVs*), being equivalent to the treated solution. The breakthrough point, indicating catalyst saturation, was identified when the *A_v_/A*_0_ ratio approached 1.

Using the relationships shown in Equations (4)–(6), the capacities of Catalysts 1 and Catalyst 2 were calculated (Table S8).

(4) $$\mathrm{Volume}\;\mathrm{to}\;\mathrm{depletion}\;\mathrm{of}\;\mathrm{catalyst}:\;v_{f}(\mathrm{mL})=\frac{A_{0}}{A_{v}}\times v$$

(5) $$\mathrm{Maximum}\;\mathrm{capacity}\;({\mathrm{mL}}_{\mathrm{NAC}}\;{{\mathrm{mL}}_{\mathrm{cat}}}^{-1})=\frac{v_{f}\;\left(mLNAC\right)}{v_{c}\;(mLCat)}$$

(6) $$\mathrm{Maximum}\;\mathrm{capacity}\;({\mathrm{mmol}}_{\mathrm{NAC}}\;{{\mathrm{mL}}_{\mathrm{cat}}}^{-1})=\frac{v_{f}\left(mLNAC\right)\times \frac{0.0001mmol}{mL}NAC}{v_{c}\;(mlLCat)}$$

where *v* (mL) is the volume of reduced NACs; *v_f_* (mL) is the final volume of reduced NACs at catalyst saturation; *v_c_* (mL) is the volume of the catalyst; *A*_0_ is the initial absorbance of an NAC; and *A_v_* is the absorbance of an NAC in effluents collected after a specified volume of reagents passed.

## 4. Conclusions

In this work, we developed amino sugar-functionalized polymeric supports for stabilizing rhenium at the sub-nanometer scale, forming highly dispersed clusters referred to as “apparent nanoparticles.” These structures provide abundant, stable active sites and enable the efficient catalytic hydrogenation of nitroaromatic compounds. A comparison of two catalysts revealed complementary performances: Catalyst 1, with higher Re loading, achieved rapid conversions in batch mode, while Catalyst 2, with its mesoporous architecture, offered superior stability and catalytic capacity under continuous flow. Importantly, this contrast demonstrates that the catalyst architecture must be tailored to the intended reactor configuration. Furthermore, the integration of these catalysts into stereolithography (SLA) 3D-printed flow reactors establishes a scalable and sustainable alternative to noble metal-based systems.

Beyond these specific results, this study highlights broader design principles: amino sugar functionalities represent a general strategy for stabilizing sub-nanometric metal species; mesoporous supports enhance long-term stability in the flow mode; and coupling tailored catalysts with additive manufacturing opens new routes for efficient, continuous, and environmentally responsible chemical production.

## Figures and Tables

**Table 1:** Surface composition (atomic %) determined by XPS.

	C			N		O			Si		Cl	Re	
Binding Energy [eV]	285.0	286.7	287.9	400.6	402.3	531.5	532.7	533.8	102.1	103.4	198.3	44.2	46.3
Compounds/Bonds	C–C, C–H	C–O–C, C–OH, C–N	C=O, O–C–O	C–NH	NH_4_^+^	O–Re, O=C O–Si	O–C, O–Si	OH, H_2_O ads.	Siloxane	SiO_2_	Cl^−^	Re^6+^ (ReO_3_)	Re^7+^ (Re_2_O_7_)
Catalyst 1	38.1	21.3	4.1	0.9	2.2	10.3	13.2	2.9	3.1	1.8	0.0	0.4	1.9
Catalyst 2	43.9	19.2	2.7	2.5	1.1	6.0	15.4	1.7	3.7	2.0	0.7	0.7	0.5

**Table 2:** Comparison of kinetic performance between Catalyst 1 and Catalyst 2 in batch-mode reductions of NACs, including turnover frequency (TOF). Reaction conditions: C_NAC_ = 0.1 mmol L^−1^; C_NaBH4_ = 0.26 mol L^−1^. The TOF values were calculated using Equation (3) based on the rhenium content measured by EDX. The calculated Re loadings correspond to **0.935 mmol Re** for Catalyst 1 (34.8 wt% Re in 0.05 g catalyst) and **0.609 mmol Re** for Catalyst 2 (22.7 wt% Re in 0.05 g catalyst).

NAC *	Catalyst	*k*_1_ ^a^ (s^−1^)	*k_1IP_* ^b^ (s^−1^)	R^2^	R_1IP_^2^	*Conversion* (%)	*Conversion t* (h)	*TOF* (h^−1^)
4-NP	1	0.841		0.992	-	91.969	0.050	1.968
	2	0.621	0.040	0.976	0.981	93.892	0.125	1.234
2,4-DNP	1	0.804		0.921	-	93.587	0.063	1.602
	2	0.533	0.145	0.948	0.968	99.229	0.138	1.186
2,4,6-TNP	1	0.676		0.963	-	97.066	0.092	1.133
	2	0.420	0.094	0.769	0.996	94.485	0.154	1.007
2-NA	1	0.605		0.932	-	85.012	0.058	1.559
	2	0.497	0.078	0.979	0.999	95.424	0.142	1.107
4-NA	1	0.263		0.998	-	93.748	0.063	1.605
	2	0.317	0.085	0.936	0.980	97.863	0.125	1.287
NB	1	1.406		0.837	-	90.652	0.029	3.326
	2	0.261	-	0.927	-	79.667	0.100	1.309

^a^ First-order rate constant (s^−1^). ^b^ First-order rate constant at induction period (s^−1^). * 4-NP: 4 nitrophenol; 2,4-DNP: 2,4-dinitrophenol; 2,4,6-TNP: 2,4,6-trinitrophenol; 2-NA: 2-nitroaniline; 4-NA: 4-nitroaniline; NB: nitrobenzene.

## Data Availability

The data are stored under permanent identifier https://doi.org/10.18150/SPJTDA.

## Supplementary Materials

The following supporting information can be downloaded at: https://www.mdpi.com/article/10.3390/molecules30183782/s1. Scheme S1: Concept of the research. Left panel: synthesis of the heterogenous catalyst. Right panel: catalytic reductions of nitroaromatic compounds, Figure S1: Synthesis of Catalysts 1 and 2: (a) matrices 1 and 2 used as supports for new catalysts, (b) washing of matrices, (c) introduction of NMDG setup, (d) heating of NMDG. Figure S2: (a) Filtration, (b) washing to remove excess amino sugars from polymer base 1, (c) reduction coupled adsorption, (d) quick test for Re saturation, (e) storage of catalysts in water for in-situ reduction of ReO_4_^−^. Figure S3: NAC (4-NP) (a) before and (b) after the addition of NaBH4, (c) Unipan peristaltic pump, (d) flow mode reduction of NACs, and (e) closer look at the reduction in the column. Figure S4: Column design displayed in Formlabs PreForm 3.3 software. Figure S5: (a) Printed column, (b) 3-D printer, washing and curing machines, (c) ready-to-use columns. Table S1: Column flow rate adjustment. Table S2: FT-IR results summary. Table S3: Water regain of polymeric matrices used for the synthesis of Catalyst 1 and Catalyst 2 (Polymer 1’ and 2’ are the replicates of polymer 1 and 2 respectively). Table S4: Surface composition of catalysts as determined by XPS, reported as both atomic % and calculated weight %. Figure S6: EDX Spectra for (a) Catalyst1 and (b) Catalyst 2. Figure S7: XRD spectra for Catalyst 1 and Catalyst 2. Figure S8: UV–Vis Spectra (on the left) and catalytic activity of Pseudo-first order models (on the right) of Catalyst 1 with different NACs (4-NP, 2,4-DNF, 2,4,6-TNF, 2-NA, 4-NA and NB) each being 0.1 mM, reacted with wet catalyst 1 of 0.05 g and 0.26 M NaBH4. Figure S9: UV–Vis Spectra (on the left) and catalytic activity of Pseudo-first order models (on the right) of catalyst 2 with different NACs (4-NP, 2,4-DNF, 2,4,6-TNF, 2-NA, 4-NA, and NB) each being 0.1 mM, reacted with wet catalyst 2 of 0.05 g and 0.26 M NaBH4. Table S5: Pseudo-first-order kinetic order results for 0.05 g of catalyst 1 with 0.1 mM NAC and 0.26 M NaBH4. Table S6: Pseudo-first-order kinetic order results for 0.05 g of catalyst 2 with 0.1 mM NAC and 0.26 M NaBH4. Table S7: Comparison of conversion rate of both Catalyst 1 and Catalyst 2. Figure S10: UV–Vis spectra of batch mode hydrogenation of mixture of NACs. Figure S11: Flow mode reduction of 4-NP (0.1 mM) with Catalyst 1. Figure S12: Flow mode reduction of 4-NP (0.1 mM) with Catalyst 2. Figure S13: Flow mode reduction of 0.1/6 mM NACs mixture (4-NP, 2,4-DNF, 2,4,6-TNF, 2- NA, 4-NA, NB) with Catalyst 2. Figure S14: HPLC-DAD chromatogram of NACs contained in the solution fed through Catalyst 2 bed. Retention times: 2,4,6-TNP: 0.690 min; 4-NP: 0.947 min; 2,4-DNP: 1.020 min; 4-NA: 1.217 min; 2-NA: 1.590 min; NB: 8.380. Figure S15: HPLC-DAD chromatogram of NACs revealed in the effluent collected from reactor containing Catalyst 2 bed. The chromatogram corresponds to the column effluent collected at BVs = 10 mL. Table S8: Capacity calculation for catalysts 1 and 2. Table S9: Integration results for the NACs mixture prior catalytic column reduction. Figure S16: XPS Survey Scans for Catalyst 1. Figure S17: XPS Survey Scans for Catalyst 2. Figure S18: C 1s spectra of (P1) Catalyst 1, and (P2) Catalyst 2. Figure S19: Re 4f spectra of (P1) Catalyst 1, and (P2) Catalyst 2. Figure S20: O 1s spectra of (P1) Catalyst 1, and (P2) Catalyst 2. Figure S21: Si 2p spectra of (P1) Catalyst 1, and (P2) Catalyst 2. Figure S22. Cl 2p spectra of (P1) Catalyst 1, and (P2) Catalyst 2.
