# Supplementary material for: Continuous Flow-Mode Synthesis of Aromatic Amines in a 3D-Printed Fixed Bed Reactor Loaded with Amino Sugar-Stabilized Re Apparent Nanoparticles
Source: Molecules. 2025 Sep 17;30(18):3782. doi: 10.3390/molecules30183782 (PMC12472570; doi:10.3390/molecules30183782)
Supplement: Supplementary file 1 [file molecules-30-03782-s001.zip › molecules-3809105-supplementary.pdf]

Supplementary materials to

Continuous Flow-Mode Synthesis of Aromatic Amines in a 3D-Printed Fixed Bed Reactor Loaded with Amino Sugar-Stabilized Re Apparent Nanoparticles

Patrick Niyirora [1], Joanna Wolska [1], Mateusz M. Marzec [2], Krystian Sokolowski [2], Anna Leśniewicz [3], Piotr Jamróz [3], Anna Dzimitrowicz [3], Andrzej Bernasik [2,4] and Piotr Cyganowski [1,*]

1 Department of Process Engineering and Technology of Polymer and Carbon Materials, Wroclaw University of Science and Technology, 27 Wybrzeze St. Wyspianskiego, 50-370 Wroclaw, Poland; niyipa05@gmail.com (P.N.); joanna.wolska@pwr.edu.pl (J.W.)

2 Academic Centre for Materials and Nanotechnology, AGH University of Krakow, Mickiewicza Av. 30, 30-059 Kraków, Poland; marzecm@agh.edu.pl (M.M.M.); krysok@agh.edu.pl (K.S.); bernasik@agh.edu.pl (A.B.)

3 Department of Analytical Chemistry and Chemical Metallurgy, Wroclaw University of Science and Technology, 27 Wybrzeze St. Wyspianskiego, 50-370 Wroclaw, Poland; anna.lesniewicz@pwr.edu.pl (A.L.); piotr.jamroz@pwr.edu.pl (P.J.); anna.dzimitrowicz@pwr.edu.pl (A.D.)

4 Faculty of Physics and Applied Computer Science, AGH University of Krakow, Mickiewicza Av. 30, 30-059 Kraków, Poland

* Correspondence: piotr.cyganowski@pwr.edu.pl; Tel.: +48-71-320-23-83

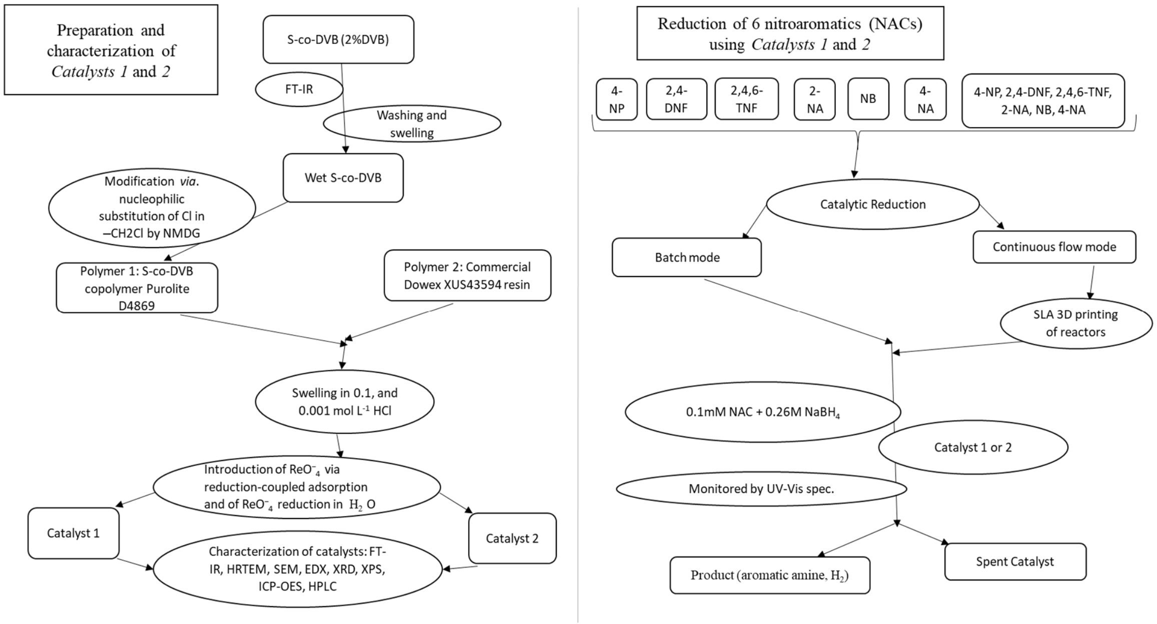

Scheme S1. Concept of the research. **Left panel**: synthesis of the heterogenous catalyst. **Right panel**: catalytic reductions of nitroaromatic compounds.

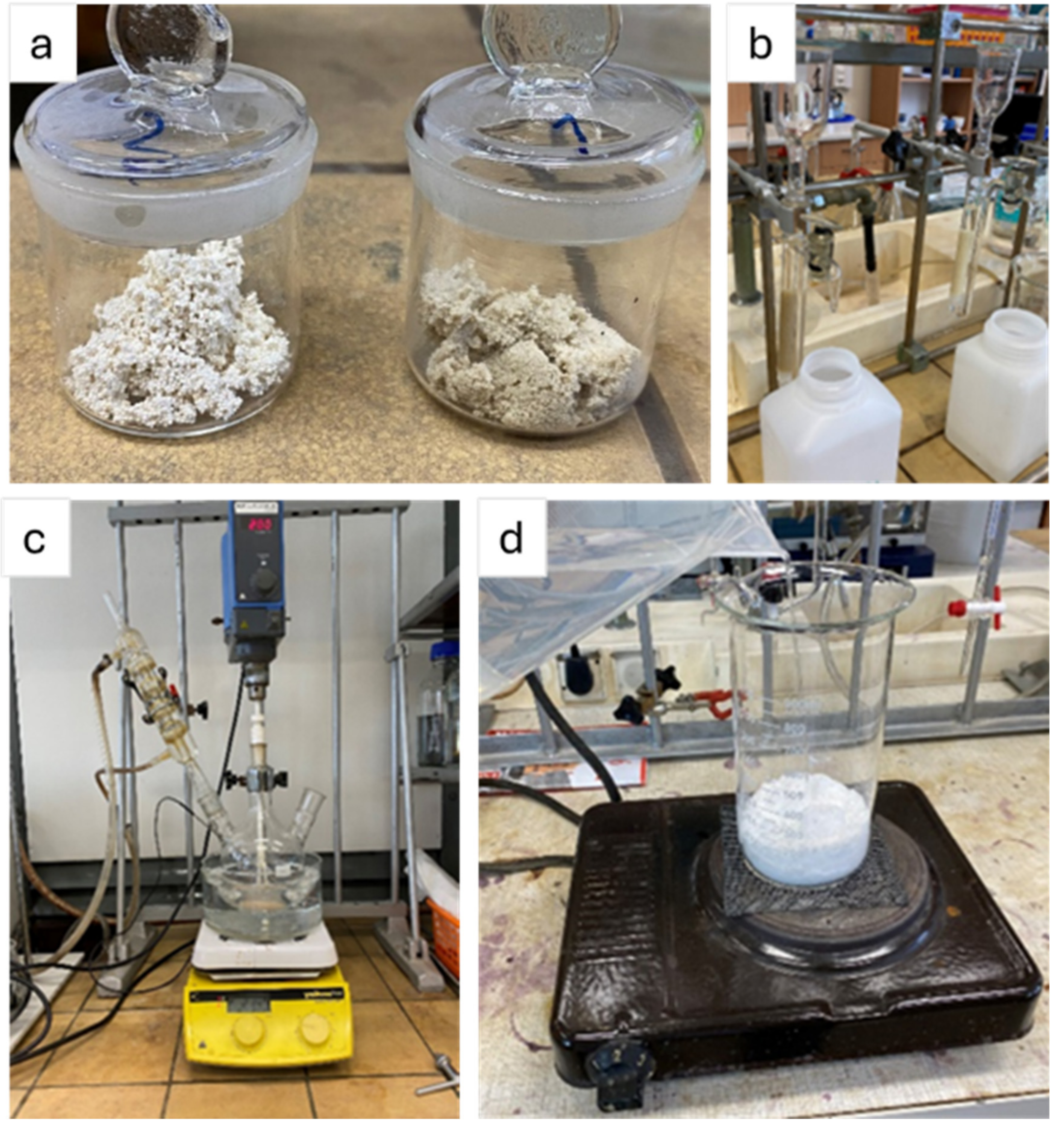

Figure S1. Synthesis of *Catalysts 1* and *2*: a) matrices 1 and 2 used as supports for new catalysts, b) washing of matrices, c) introduction of NMDG setup, d) heating of NMDG.

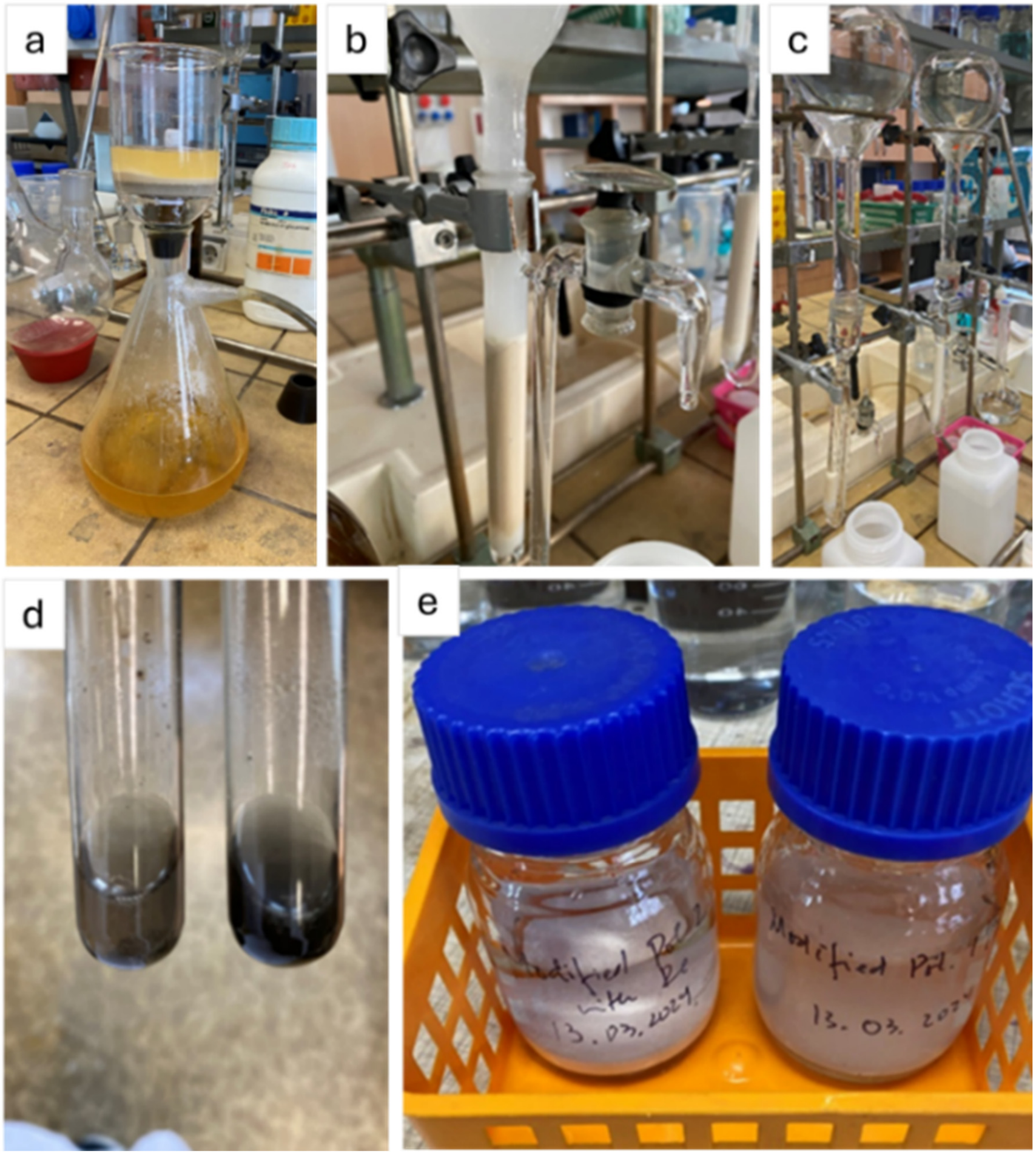

Figure S2. a) Filtration, b) washing to remove excess amino sugars from polymer base 1, c) reduction coupled adsorption, d) quick test for Re saturation, e) storage of catalysts in water for in-situ reduction of ReO_4^-.

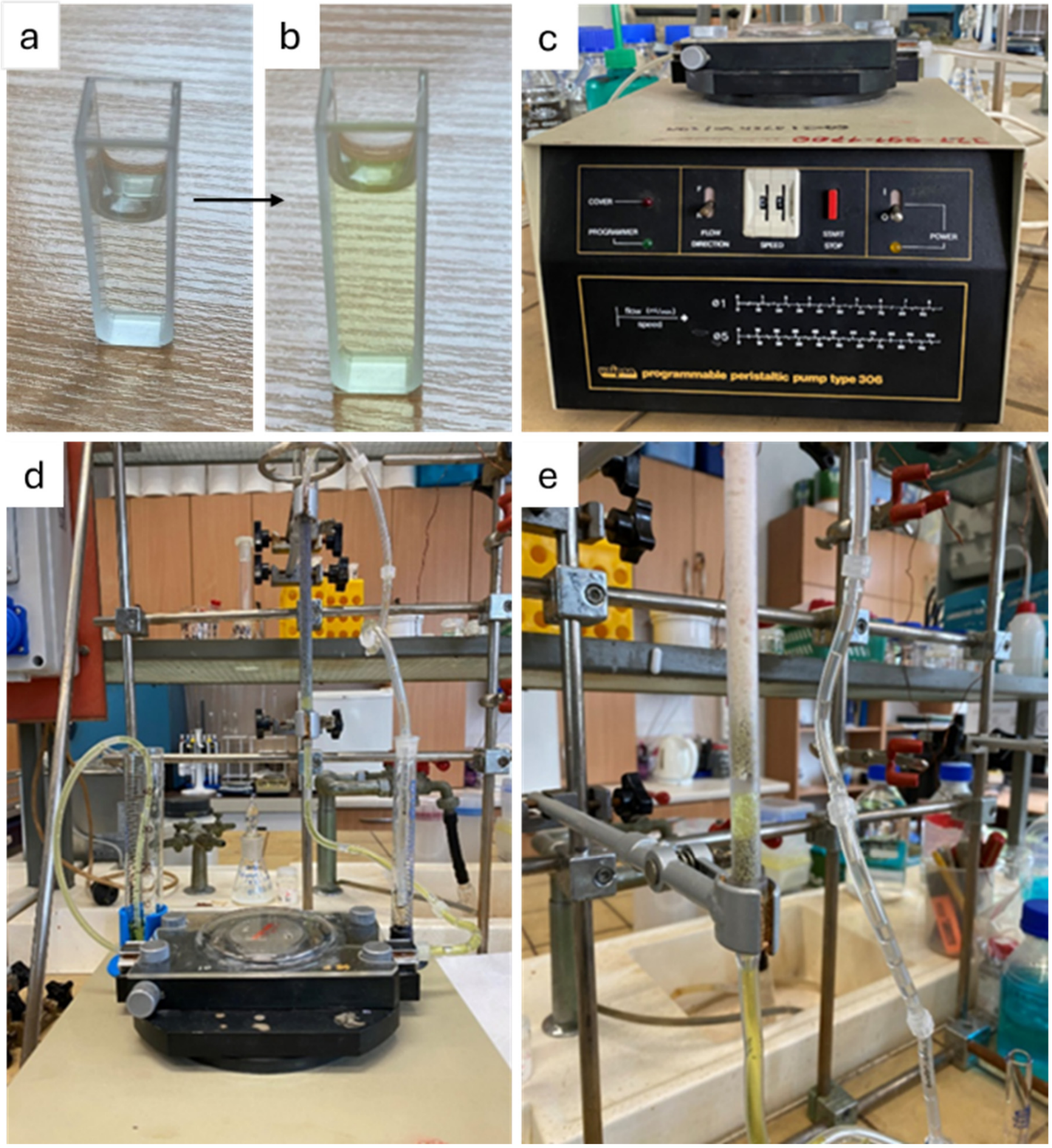

Figure S3. NAC (4-NP) a) before and b) after the addition of $NaBH_4$, c) Unipan peristaltic pump, d) flow mode reduction of NACs, and e) closer look at the reduction in the column.

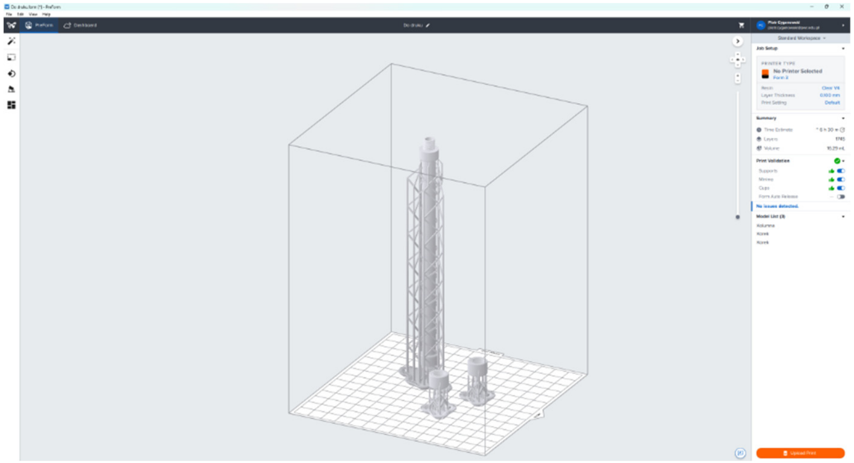

Figure S4. Column design displayed in Formlabs PreForm software.

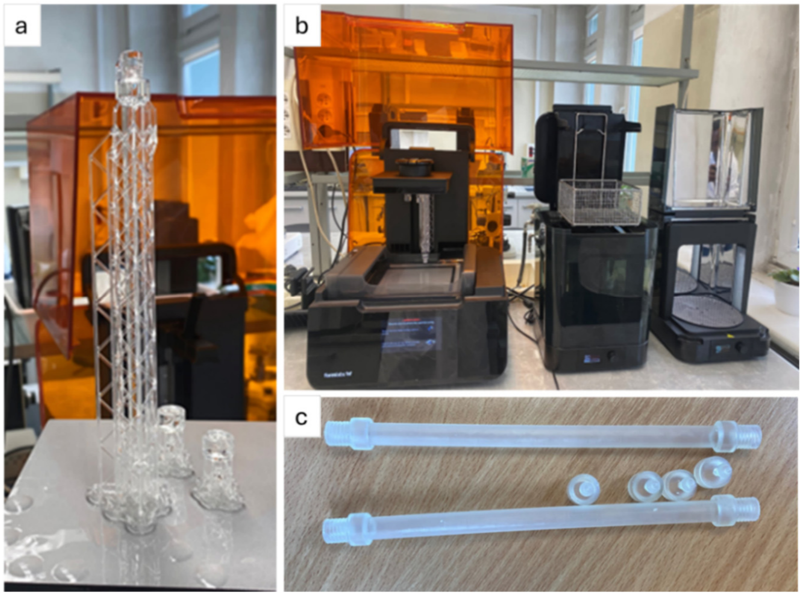

Figure S5. a) Printed column, b) 3-D printer, washing and curing machines, c) ready-to-use columns.

Table S1. Column flow rate adjustment

Speed	Time (min)		Volume (mL)	Flow rate (mL min^{-1})
2	8		4.0	0.5
5	4		5.0	1.25
10	2		5.0	2.5
20	1		5.0	5.0
30	1		7.5	7.5
40	1		10.0	10

Table S2. FT-IR results summary.

**Interactions**	**Characteristic Band Location/Shift (cm^{-1})**			**Possible explanation**
	**Polymer base 1**	**Resin 1**	**Resin 2**	
C-Cl	554<br>671	-	-	*A decrease or disappearance indicates the removal of chloro-methylene groups* [51].
C=C bending	822	888	890	
C=C stretching in aromatic rings (likely from styrene)	1509	1394<br>1419	1386<br>1393	
O=C=O stretching	-	2363	2360	
C-H stretching	2920	2979	2979	
N-H and O-H stretching	-	3367	3365<br>3349	*New peaks suggest the introduction of amine groups* [51].
O-H stretching	-	1073	1073	*Polyol moieties from NMDG* [51].

Resins' Water Regain Calculation.

Following the drying of centrifuged catalysts samples, the H_2O regain for these resins was calculated using the formula in Equation S1 and the results are shown in Table S3.

$$W_r = \frac{m_w - m_d}{m_d} \qquad \textit{Equation S1}$$

W_r = Water Regain

m_w = mass of hydrated polymer

m_d = mass of dry polymer

Table S3. Water regain of polymeric matrices used for the synthesis of *Catalyst 1* and *Catalyst 2* (Polymer 1' and 2' are the replicates of polymer 1 and 2 respectively).

	Wet weight (g)	Dry weight (g)	Wr (gH_2O g dry polymer^{-1})
Polymer 1	0.311	0.389	0.367
Polymer 1'	0.545	0.398	0.369
Polymer 2	0.538	0.294	0.832
Polymer 2'	0.567	0.319	0.780

To enable comparison between different techniques (XPS, ICP-OES, EDX), atomic percentages derived from XPS analysis were recalculated to weight percentages using Eq. 8, where M_i is the atomic mass of element i and at.% is the atomic percentage measured by XPS:

$$\boldsymbol{wt.\%i} = \frac{(\boldsymbol{at.}\%\boldsymbol{i} \times \boldsymbol{Mi})}{\boldsymbol{\Sigma}\,(\boldsymbol{at.}\% \times \boldsymbol{M})} \qquad \text{(S2)}$$

Table S4. Surface composition of catalysts as determined by XPS, reported as both atomic % and calculated weight %.

**Element**	**Binding Energy [eV]**	**Compound/Bond**	**Catalyst 1 (at. %)**	**Catalyst 1 (wt. %)**	**Catalyst 2 (at. %)**	**Catalyst 2 (wt. %)**
C	285.0–287.9	C–C, C–O, C=O	38.1	22.4	43.9	25.8
N	400.6–402.3	C–NH, NH_4^+	21.3	17.9	19.2	16.3
O	531.5–533.8	O–Re, O–C, –OH	4.1 + 13.2 + 2.9	18.7	2.7 + 15.4 + 1.7	17.2
Si	102.1–103.4	Si–O, SiO_2	3.1 + 1.8	11.5	3.7 + 2.0	12.1
Cl	198.3	Cl^-	0.0	–	0.7	2.3
Re	44.2–46.3	Re^{6+}, Re^{7+}	2.3	29.5	1.2	26.3

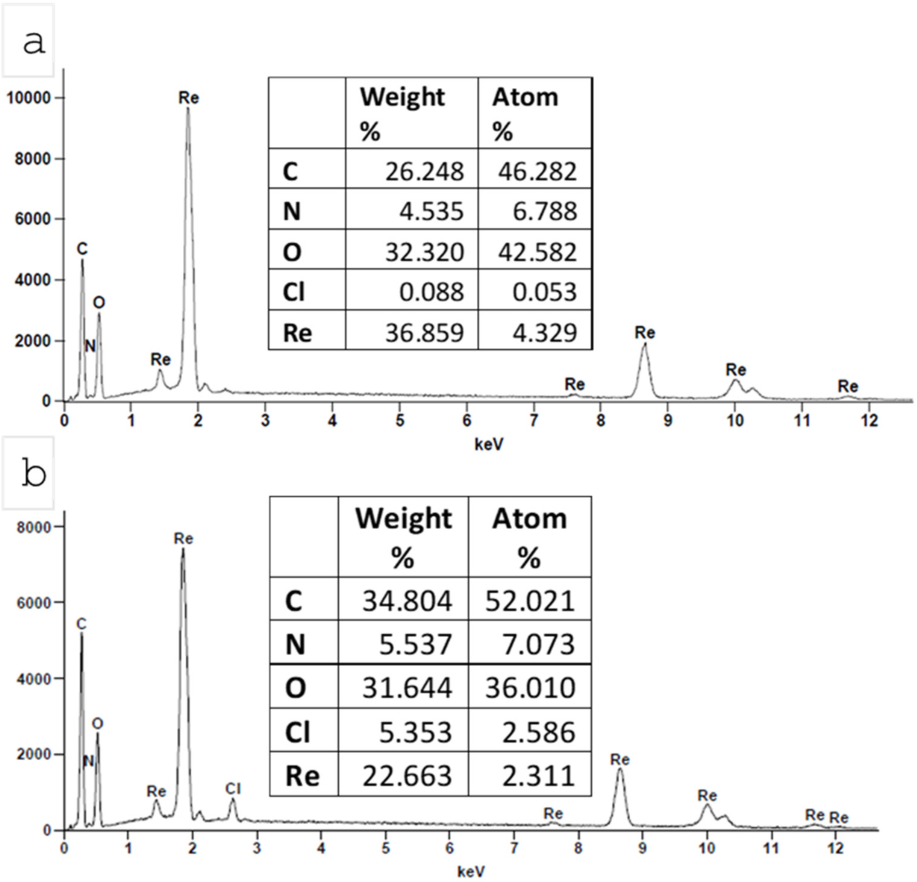

a	Weight %	Atom %
C	26.248	46.282
N	4.535	6.788
O	32.320	42.582
Cl	0.088	0.053
Re	36.859	4.329

b	Weight %	Atom %
C	34.804	52.021
N	5.537	7.073
O	31.644	36.010
Cl	5.353	2.586
Re	22.663	2.311

Figure S6. EDX Spectra for a) *Catalyst 1* and b) *Catalyst 2*.

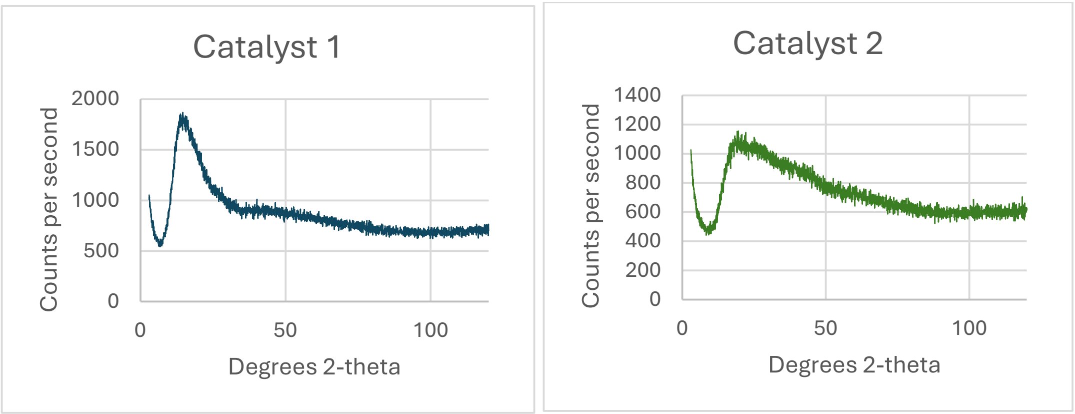

Figure S7. XRD spectra for *Catalyst 1* and *Catalyst 2*.

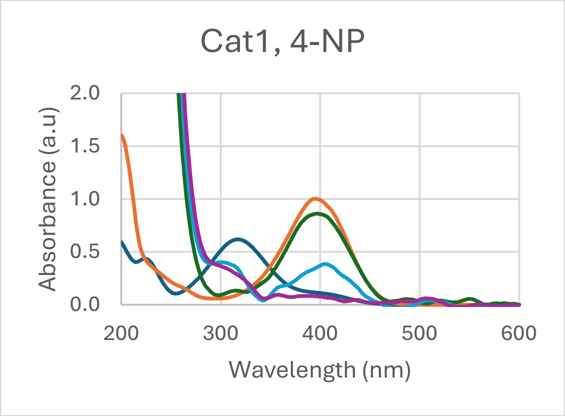
Cat1, 4-NP
Absorbance (a.u)
2.0
1.5
1.0
0.5
0.0
200 300 400 500 600
Wavelength (nm)

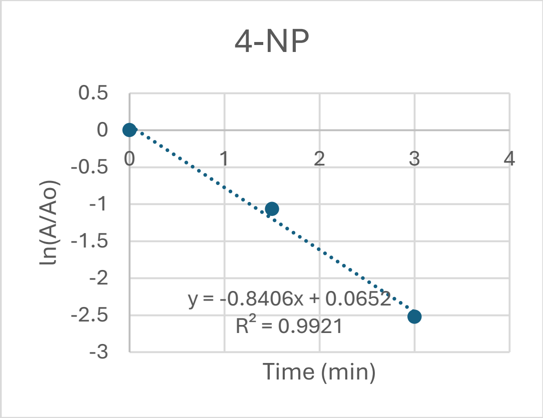
4-NP
ln(A/Ao)
0.5
0
-0.5
-1
-1.5
-2
-2.5
-3
0 1 2 3 4
y = -0.8406x + 0.0652
R² = 0.9921
Time (min)

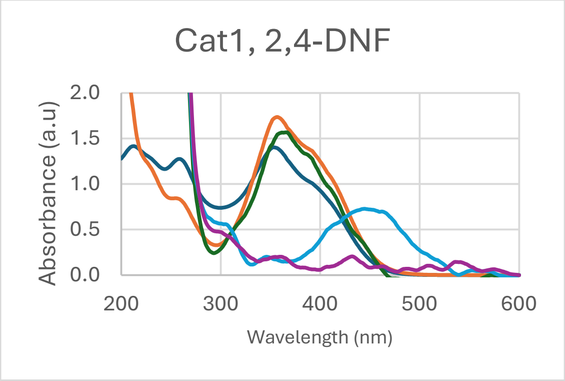
Cat1, 2,4-DNF
Absorbance (a.u)
2.0
1.5
1.0
0.5
0.0
200 300 400 500 600
Wavelength (nm)

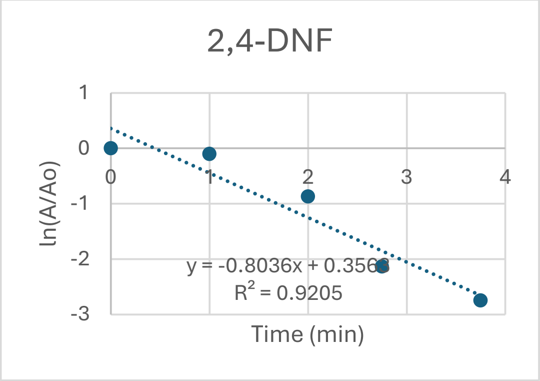
2,4-DNF
ln(A/Ao)
1
0
-1
-2
-3
0 1 2 3 4
y = -0.8036x + 0.3568
R² = 0.9205
Time (min)

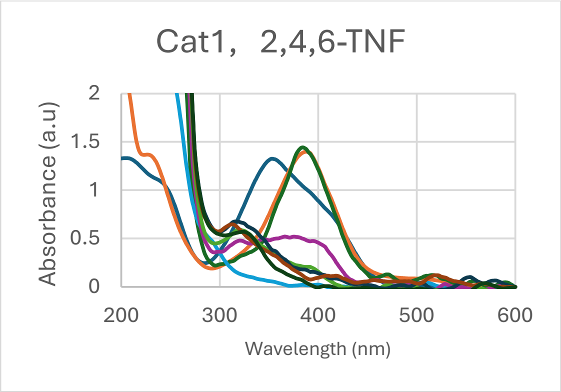
Cat1, 2,4,6-TNF
Absorbance (a.u)
2
1.5
1
0.5
0
200 300 400 500 600
Wavelength (nm)

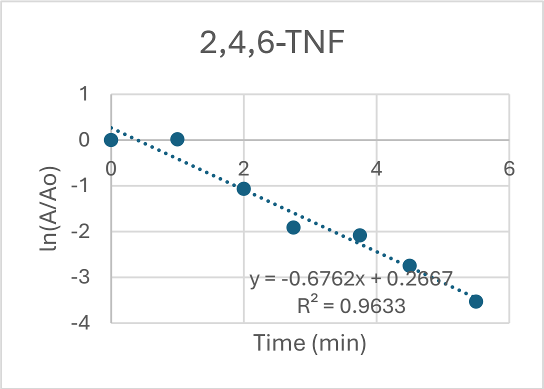
2,4,6-TNF
ln(A/Ao)
1
0
-1
-2
-3
-4
0 2 4 6
y = -0.6762x + 0.2667
R² = 0.9633
Time (min)

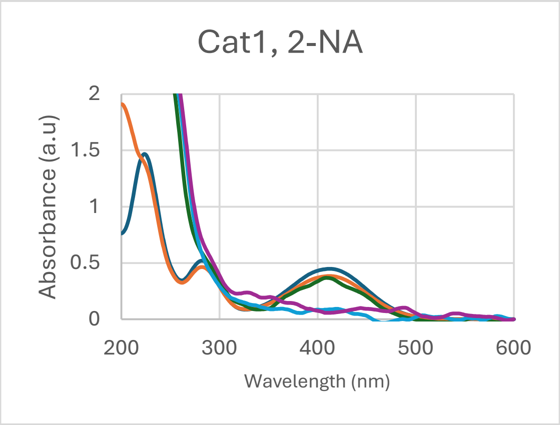
Cat1, 2-NA
Absorbance (a.u)
2
1.5
1
0.5
0
200 300 400 500 600
Wavelength (nm)

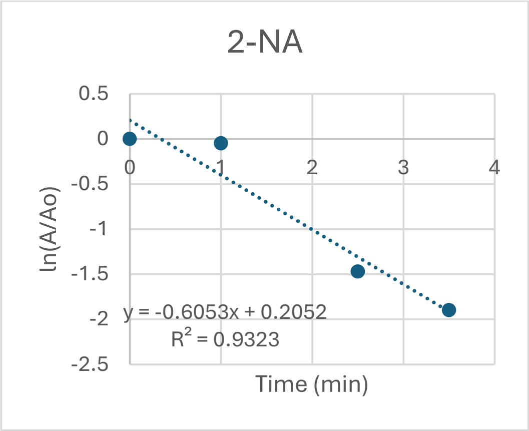
2-NA
ln(A/Ao)
0.5
0
-0.5
-1
-1.5
-2
-2.5
0 1 2 3 4
y = -0.6053x + 0.2052
R² = 0.9323
Time (min)

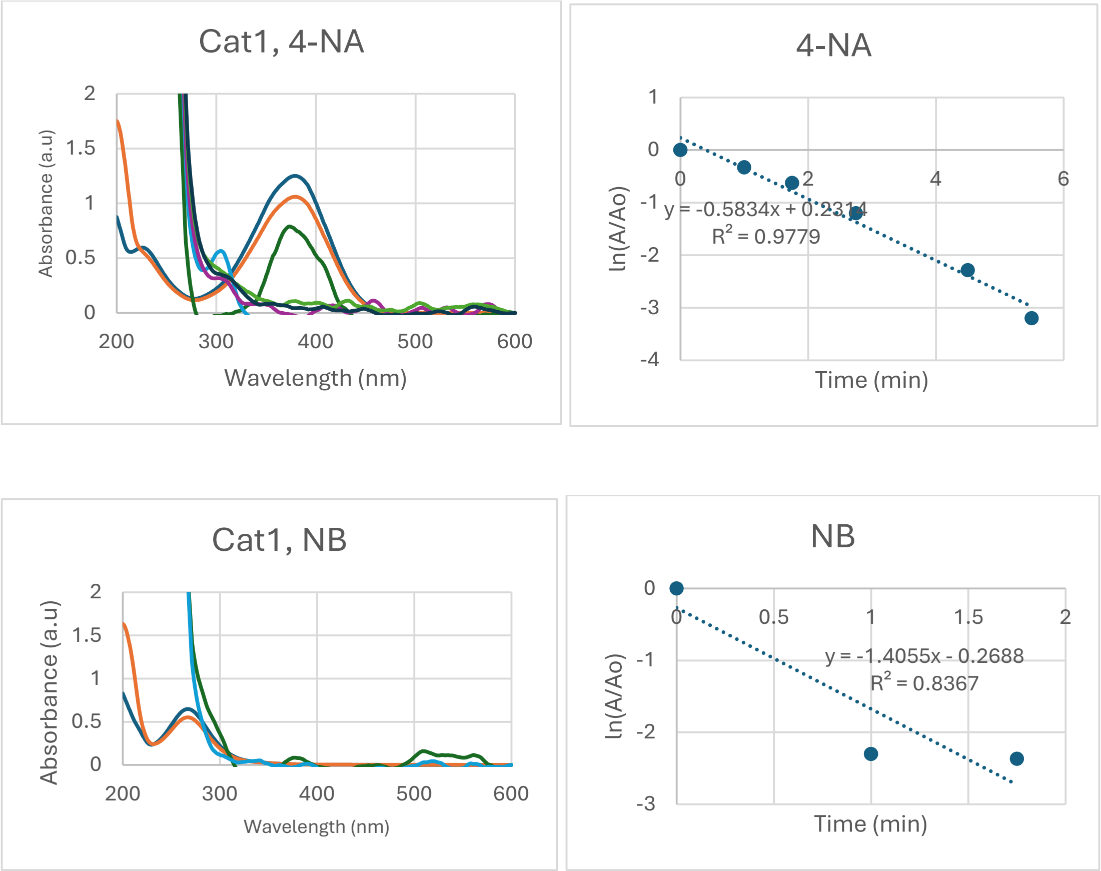

Figure S8. UV-Vis Spectra (on the left) and catalytic activity of Pseudo-first order models (on the right) of Catalyst 1 with different NACs (4-NP, 2,4-DNF, 2,4,6-TNF, 2-NA, 4-NA and NB) each being 0.1 mM, reacted with wet catalyst 1 of 0.05 g and 0.26 M $NaBH_4$.

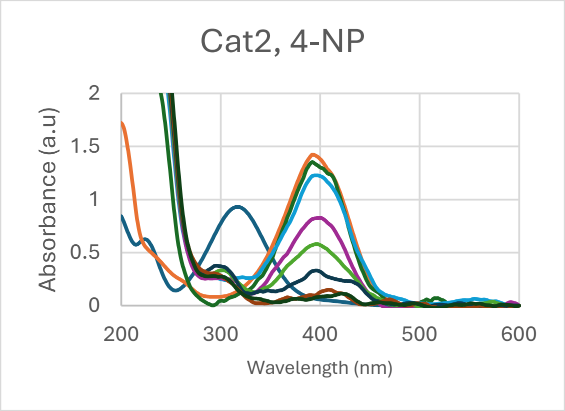
Cat2, 4-NP
Absorbance (a.u)
2
1.5
1
0.5
0
200
300
400
500
600
Wavelength (nm)

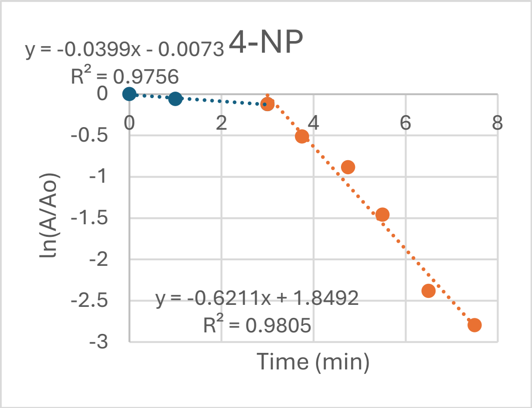
4-NP
y = -0.0399x - 0.0073
R² = 0.9756
y = -0.6211x + 1.8492
R² = 0.9805
ln(A/Ao)
0
-0.5
-1
-1.5
-2
-2.5
-3
0
2
4
6
8
Time (min)

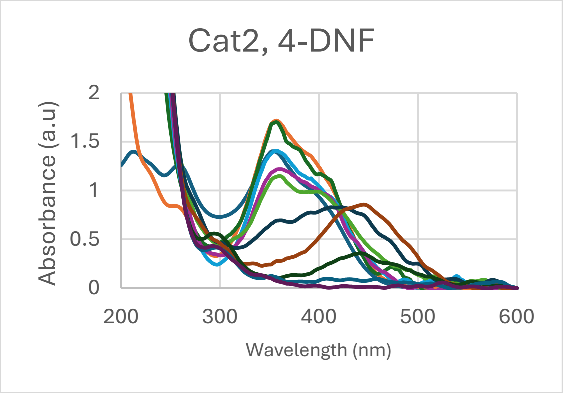
Cat2, 4-DNF
Absorbance (a.u)
2
1.5
1
0.5
0
200
300
400
500
600
Wavelength (nm)

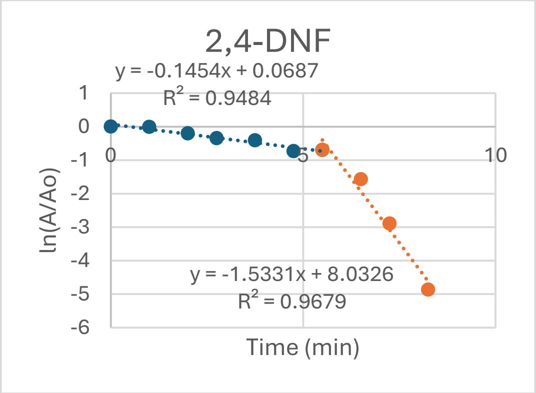
2,4-DNF
y = -0.1454x + 0.0687
R² = 0.9484
y = -1.5331x + 8.0326
R² = 0.9679
ln(A/Ao)
1
0
-1
-2
-3
-4
-5
-6
0
5
10
Time (min)

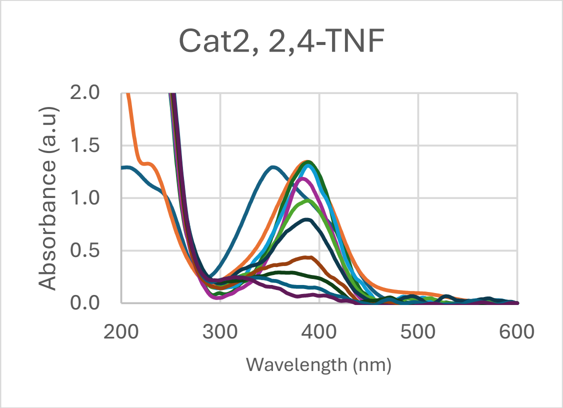
Cat2, 2,4-TNF
Absorbance (a.u)
2.0
1.5
1.0
0.5
0.0
200
300
400
500
600
Wavelength (nm)

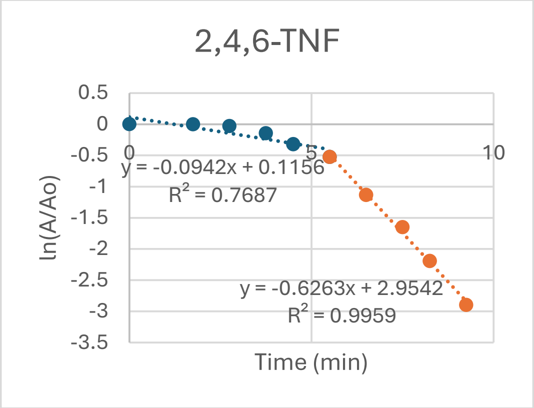
2,4,6-TNF
y = -0.0942x + 0.1156
R² = 0.7687
y = -0.6263x + 2.9542
R² = 0.9959
ln(A/Ao)
0.5
0
-0.5
-1
-1.5
-2
-2.5
-3
-3.5
0
5
10
Time (min)

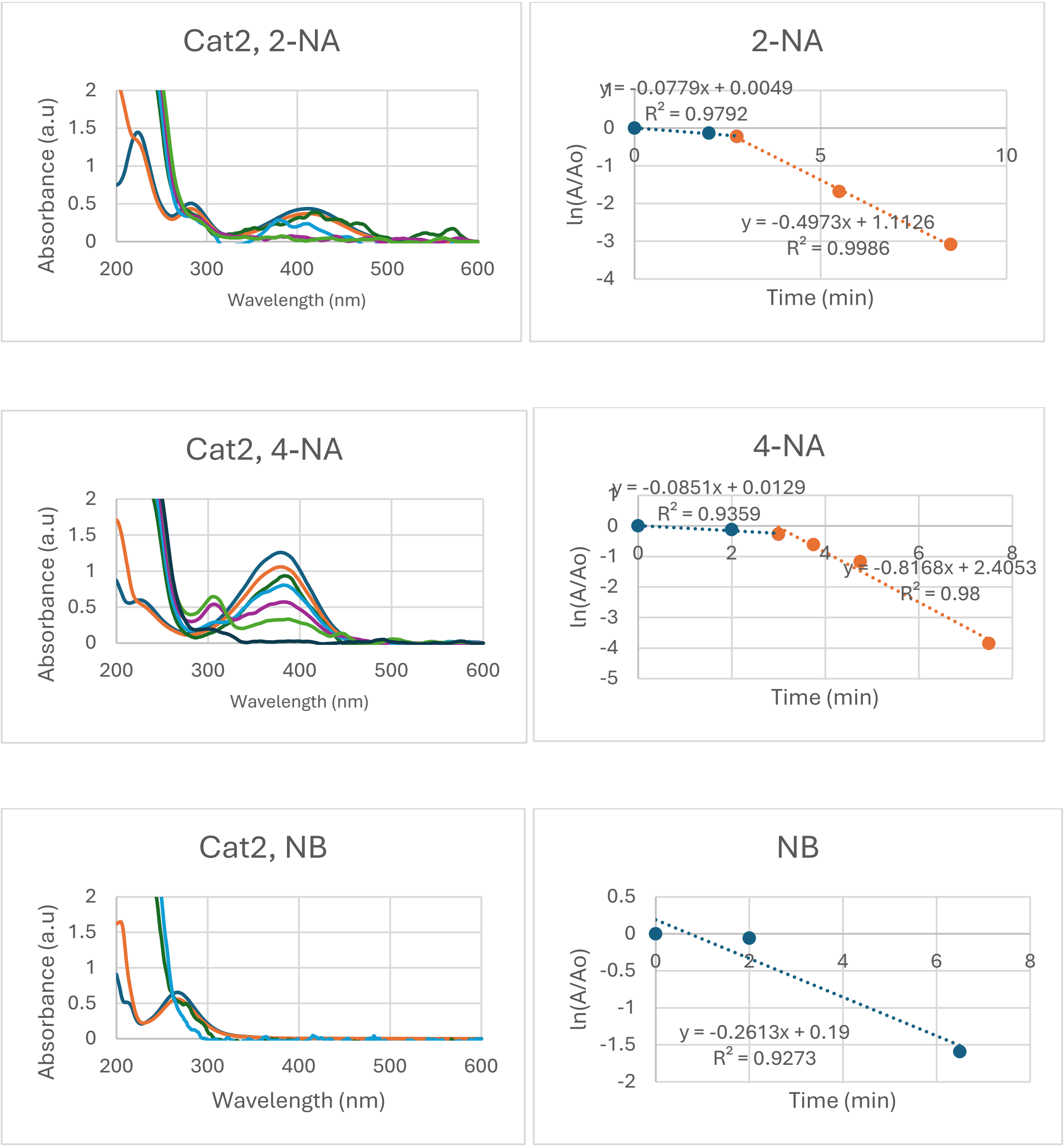

Figure S9. UV–Vis Spectra (on the left) and catalytic activity of Pseudo-first order models (on the right) of catalyst 2 with different NACs (4-NP, 2,4-DNF, 2,4,6-TNF, 2-NA, 4-NA, and NB) each being 0.1 mM, reacted with wet catalyst 2 of 0.05 g and 0.26 M $NaBH_4$

Table S5 and Table S6 present the kinetic parameters, reaction efficiency, and conversion times for six nitroaromatic compounds (NACs), including their rate constants *(k_1)*, correlation coefficients (R^2), conversion rates, and reaction times (t)

Table S5. Pseudo-first-order kinetic order results for 0.05 g of catalyst 1 with 0.1 mM NAC and 0.26 M $NaBH_4$.

**NACs name**	**k_1 (s^{-1})**	**R^2**	**Conversion**	**t (s)**
4-NP	0.841	0.992	0.920	180
2,4-DNP	0.804	0.921	0.936	225
2,4,6 TNP	0.676	0.963	0.971	330
2-NA	0.605	0.932	0.850	210
4-NA	0.263	0.998	0.937	225
NB	1.406	0.837	0.907	105

k_1 is the rate constant of the reaction, t (sec) is the conversion time, and R^2 is the coefficient of determination.

Table S6. Pseudo-first-order kinetic order results for 0.05 g of catalyst 2 with 0.1 mM NAC and 0.26 M $NaBH_4$.

**NACs name**	**k_1 (s^{-1})**	**k_1' (s^{-1})**	**R^2**	**R^{2}'**	**Conversion**	**t (s)**
4-NP	0.040	0.621	0.976	0.981	0.939	450
2,4-DNP	0.145	1.533	0.948	0.968	0.992	495
2,4,6 TNP	0.094	0.626	0.769	0.996	0.945	555
2-NA	0.078	0.497	0.979	0.999	0.954	510
4-NA	0.085	0.817	0.936	0.980	0.979	450
NB	0.261		0.927		0.797	360

k_1 is the rate constant of the reaction, t is the conversion time, and R^2 is the coefficient of determination.

To compare the conversion of all NACs, the conversion rate (min^{-1}) was calculated, and the results are shown in Table S7 for both catalysts.

Table S7. Comparison of conversion rate of both Catalyst 1 and Catalyst 2.

**NACs name**	**Conversion rate (min^{-1})**	
	**Catalyst1**	**Catalyst 2**
4-NP	0.307	0.125
2,4-DNP	0.250	0.120
2,4,6 TNP	0.176	0.102
2-NA	0.243	0.112
4-NA	0.250	0.130
NB	0.518	0.133

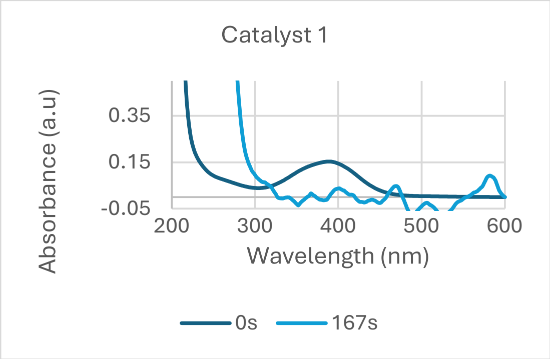

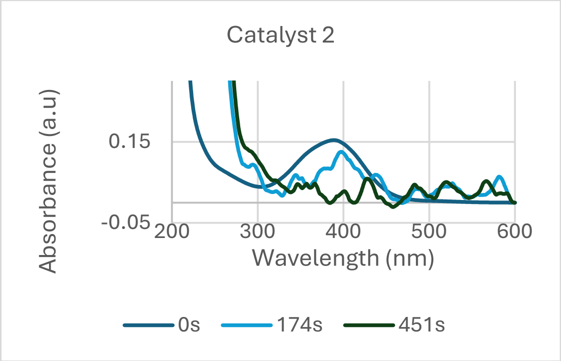

Figure S10. UV–Vis spectra of batch mode hydrogenation of mixture of NACs.

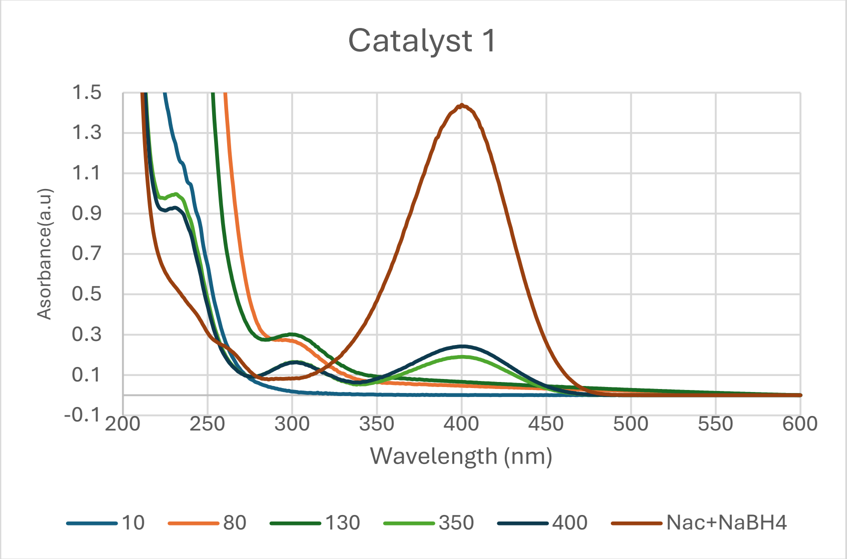

Figure S11. Flow mode reduction of 4-NP (0.1 mM) with Catalyst 1.

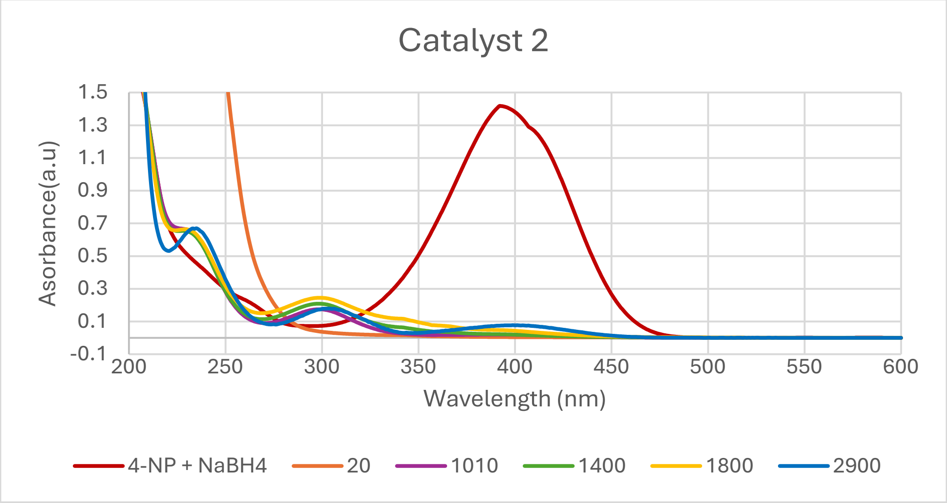

Figure S12. Flow mode reduction of 4-NP (0.1 mM) with Catalyst 2.

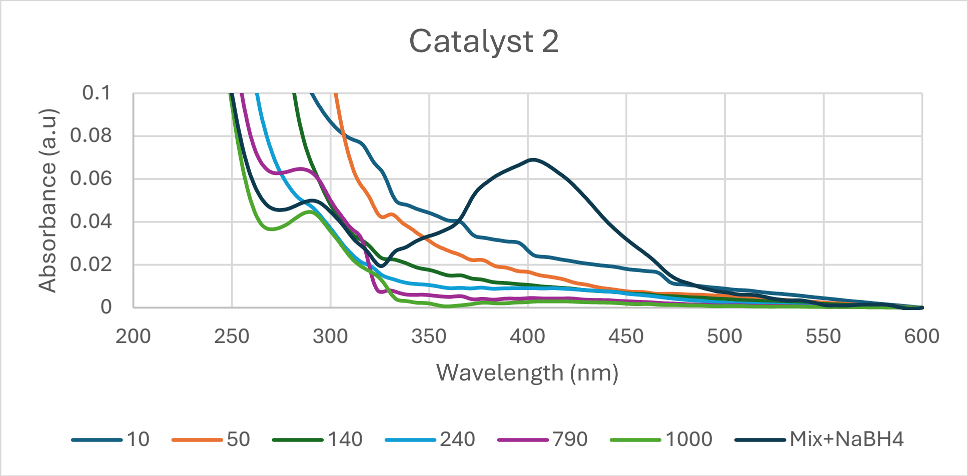

Figure S13. Flow mode reduction of 0.1/6 mM NACs mixture (4-NP, 2,4-DNF, 2,4,6-TNF, 2- NA, 4-NA, NB) with Catalyst 2.

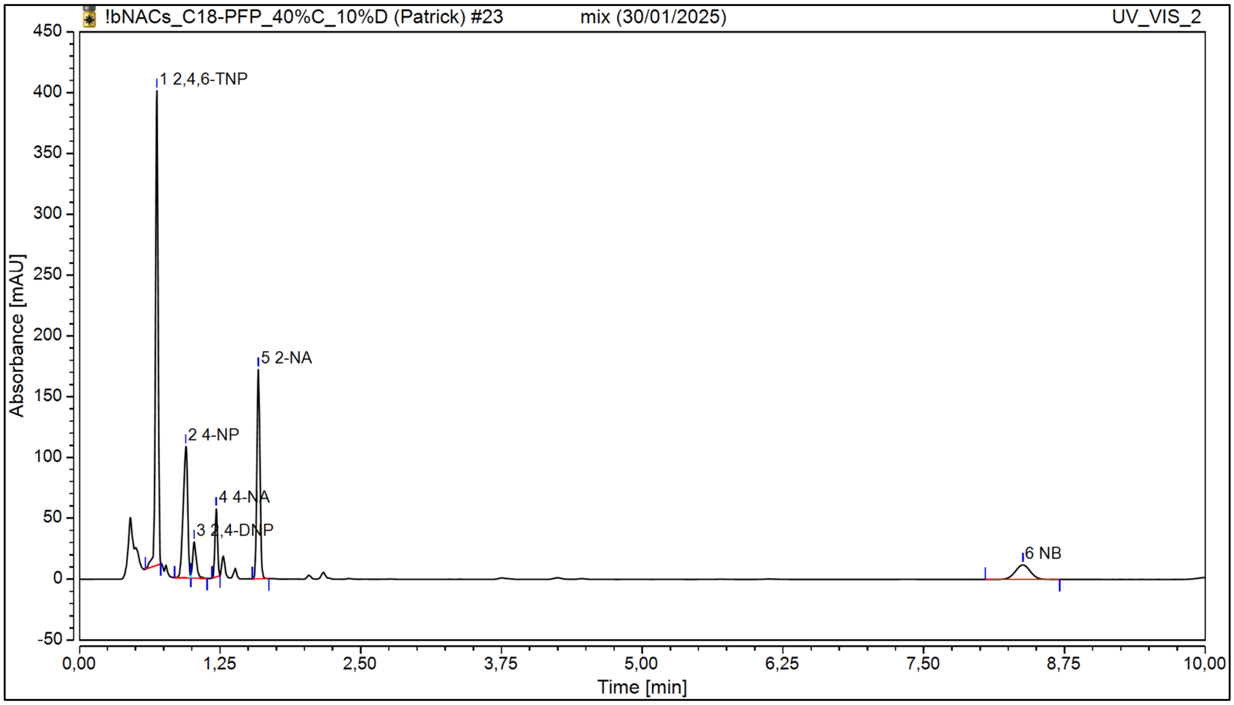

Figure S14. HPLC-DAD chromatogram of NACs contained in the solution fed through Catalyst 2 bed. Retention times: 2,4,6-TNP: 0.690 min; 4-NP: 0.947 min; 2,4-DNP: 1.020 min; 4-NA: 1.217 min; 2-NA: 1.590 min; NB: 8.380.

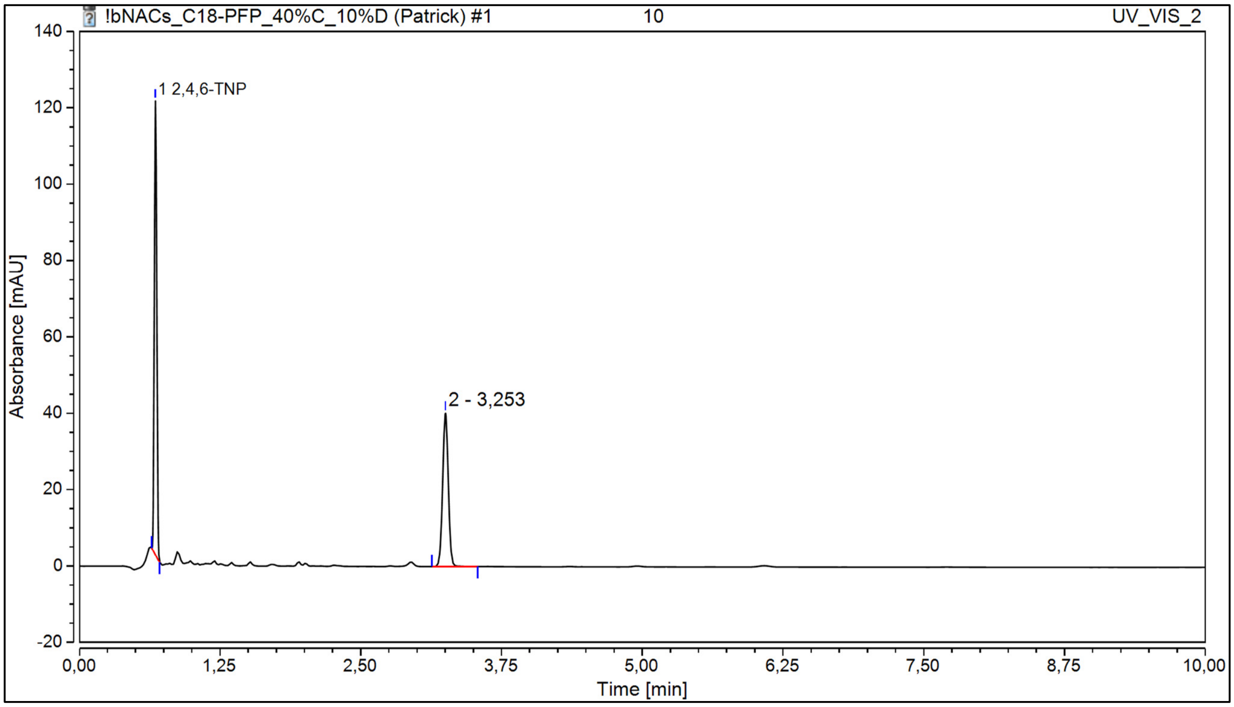

Figure S15. HPLC-DAD chromatogram of NACs revealed in the effluent collected from reactor containing Catalyst 2 bed. The chromatogram corresponds to the column effluent collected at BVs=10 mL.

Catalysts Capacity Calculation

Using the ratio of final absorbance (At) to initial absorbance (Ao) as shown in Equations S3 - S6, the capacity calculations for catalysts 1 and 2 based on absorbance measurements at a wavelength of 400 nm are shown in Table 8 below.

Table S8. Capacity calculation for Catalysts 1 and 2.

**Catalyst**	**A_o (a.u)**	**A_v (a.u)**	**v (mL)**	**A_v/A_o**	**v_f (mL)**	**Catalyst volume, v_c (mL)**	**Capacity ($ml_{NAC}ml_{cat}^{-1}$)**	**Capacity ($mmol_{NAC}ml_{cat}^{-1}$)**
**1**	1.440	0.168	400	0.117	3432.2	1.0	3432.2	0.343
**2**	1.381	0.076	2900	0.055	52569.1	3.8	13834.0	1.383

where:

- C_0 (mol) is the initial concentration of 4-nitrophenol
- C_v (mol) is the concentration of 4-nitrophenol at volume v
- v (mL) is the volume of reduced 4-nitrophenol
- A_0 is the initial absorbance of 4-nitrophenol
- A_t is the absorbance of 4-nitrophenol at time t

$$\frac{At}{Ao} = \frac{Cv}{Co} \qquad \textit{Equation S3}$$

$$\textit{Volume to depletion of catalyst, } v_f\,(\text{mL}) = \frac{Ao}{Av} * v \qquad \textit{Equation S4}$$

$$\textit{Maximum capacity } (\text{mL}_{NAC}\ \text{mL}_{cat}^{-1}) = \frac{vf\ (ml\ NAC)}{vc\ (ml\ Cat)} \qquad \textit{Equation S5}$$

$$\textit{Maximum capacity } (\text{mmoL}_{NAC}\ \text{mL}_{cat}^{-1}) = \frac{vf\ (mL\ NAC) \times \frac{0.0001mmol}{mL} NAC}{vc\ \ (mL\ Cat)} \qquad \textit{Equation S6}$$

The amount of NACs in the mixture [mM] was calculated from relative area using the formula:

$$C_{NAC} = C_{total} \times \frac{Relative\ area}{100} \quad equation\ S7$$

C_{total}= 0.1 mM

Relative Area = Relative Area [%] from the table S9

Table S9. Integration results for the NACs mixture prior catalytic column reduction.

No.	Peak name	Retention Time [min]	Area [mAUmin]	Height [mAU]	Relative Area [%]	Relative Height [%]	Amount [mM]
1	2,4,6-TNP	0.690	10.5946	390.216	41.54	50.81	0.0415
2	4-NP	0.947	4.9601	108.173	19.45	14.09	0.0194
3	2,4-DNP	1.020	1.1341	29.761	4.45	3.88	0.0044
4	4-NA	1.217	1.4871	55.726	5.83	7.26	0.0058
5	2-NA	1.590	5.4754	172.130	21.47	22.41	0.0215
6	NB	8.380	1.8521	11.940	7.26	1.55	0.0073
**Total:**			**25.503**	**767.945**	**100.000**	**100.000**	0.1000

XPS Analysis

Experimental

The XPS analyses were carried out in a PHI VersaProbeII Scanning XPS system using monochromatic Al Kα (1486.6 eV) X-rays focused to a 100 μm spot and scanned over the area of 400 μm × 400 μm. The photoelectron take-off angle was 45° and the pass energy in the analyzer was set to 117.50 eV (0.5 eV step) for survey scans and 46.95 eV (0.1 eV step) to obtain high-energy resolution spectra for the C 1s, O 1s, Re 4f, Si 2p and Cl 2p regions. A dual beam charge compensation with 7 eV Ar^+ ions and 1 eV electrons were used to maintain a constant sample surface potential regardless of the sample conductivity. All XPS spectra were charge referenced to the unfunctionalized, saturated carbon (C–C) C1s peak at 285.0 eV. The operating pressure in the analytical chamber was less than $3x10^{-9}$ mbar. Deconvolution of spectra was carried out using PHI MultiPak software (v.9.9.3). Spectrum background was subtracted using the Shirley method.

Results

Surface concentrations of chemical bonds obtained from fitting XPS data for both samples are listed in Table 1.

The C 1s spectra for both analyzed samples were fitted with three lines: the first line at 285.0 eV indicates C-C aliphatic bonds, the second line at 286.7 eV originate from C–O and/or C–OH and/or bonds C–N bonds, and the last line found at 287.9 eV indicates presence of C=O and/or O–C–O bonds in the sample [1, 2].

The O 1s spectra were fitted with three components of which the first line at 531.5 eV indicates oxygen in metal oxides (O–Re) and/or O=C functional groups, second line at 532.7 eV indicates the presence of O–C and/or O–Si bonds and the last line at 533.8 eV indicate –OH type compounds and/or adsorbed water [3-5].

The Re 4f region was fitted using two doublet structures (double separation $f_{7/2} - f_{5/2}$ equals 2.4 eV) in which the first $4f_{7/2}$ line centered at 44.2 eV is assigned to Re^{6+} oxidation state like in ReO_3, and the second $4f_{7/2}$ line found at 46.3 eV comes from Re^{7+} oxidation state like in Re_2O_7 [4].

The N 1s spectra were fitted with two lines, the first one at 400.6 eV indicating presence of C–NH bonds, and the second line at 402.3 eV indicating presence of NH_4^+ ions [3, 4].

The Cl 2p spectra were fitted with one doublet structure (doublet separation $p_{3/2} - p_{1/2}$ equals 1.6 eV) with the main $2p_{3/2}$ line centered at 198.3 eV which points out the existence of Cl^- ions in chlorides [4].

The Si 2p spectra were fitted using two doublet structures (double separation $p_{3/2}$ - $p_{1/2}$ equals 0.61 eV) in which the first $2p_{3/2}$ line at 102.1 eV shows the presence of Si-O bonds in silicone and/or siloxane compounds, and second $2p_{3/2}$ line at 103.4 eV shows presence of silica [4].

References

[1] High Resolution XPS of Organic Polymers: The Scienta ESCA300 Database (Beamson, G.; Briggs, D.), Journal of Chemical Education, 70 (1993) A25.
[2] P. Rouxhet, M. Genet, XPS analysis of bio-organic systems, Surface and Interface Analysis, 43 (2011) 1453-1470.

[3] M.J. Genet, C.C. Dupont-Gillain, P.G. Rouxhet, XPS Analysis of Biosystems and Biomaterials, in: E. Matijevic (Ed.) Medical Applictions of Colloids, Springer Science Business Media, LLC, 2008.
[4] A.V. Naumkin, A. Kraut-Vass, S.W. Gaarenstroom, C.J. Powell, NIST X-ray Photoelectron Spectroscopy Database Version 5.0, in, http:/srdata.nist.gov/xps/, 2023.
[5] D. Briggs, Surface Analysis of Polymers by XPS and Static SIMS, Cambridge University Press, New York, 2005.

Survey scans

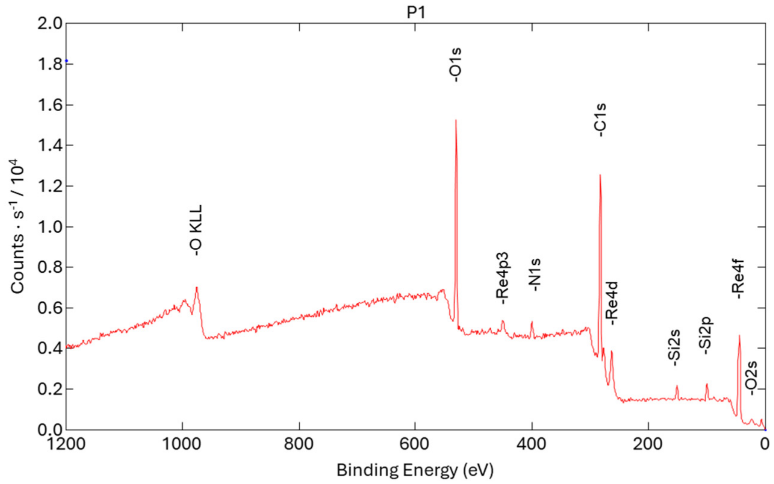

Figure S16. XPS Survey Scans for Catalyst 1.

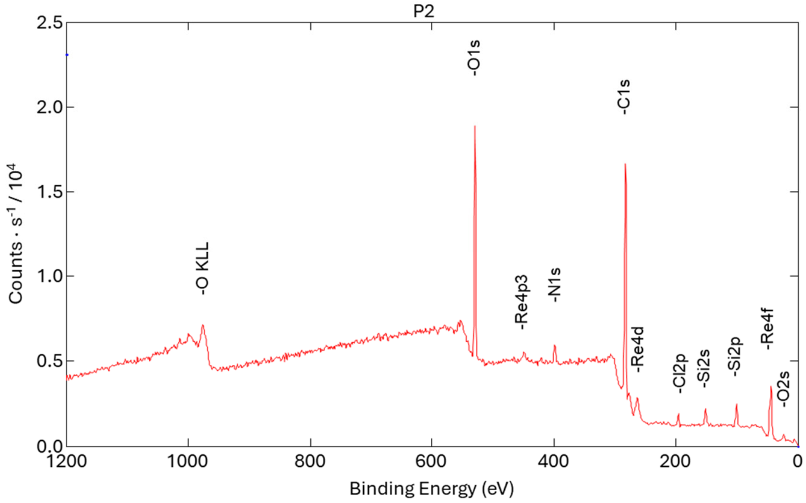

Figure S17.XPS Survey Scans for Catalyst 2.

High resolution spectra and analysis

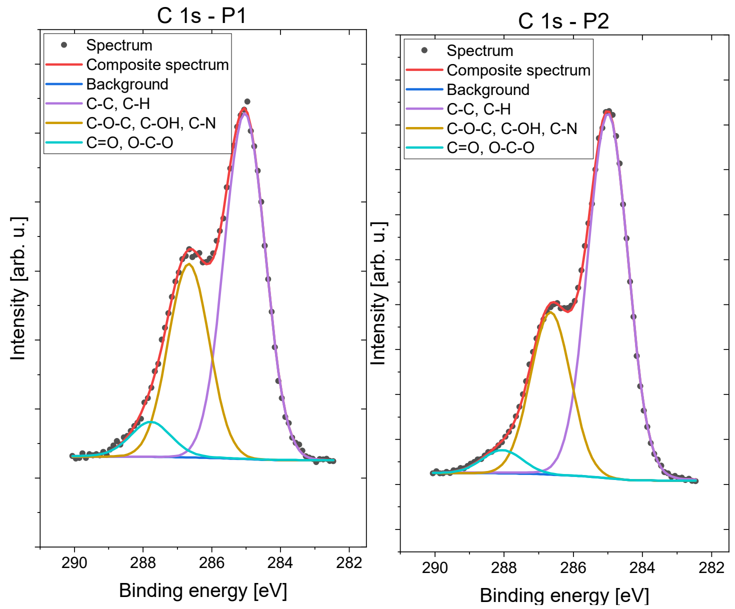

Figure S18. C 1s spectra of (P1) Catalyst 1, and (P2) Catalyst 2.

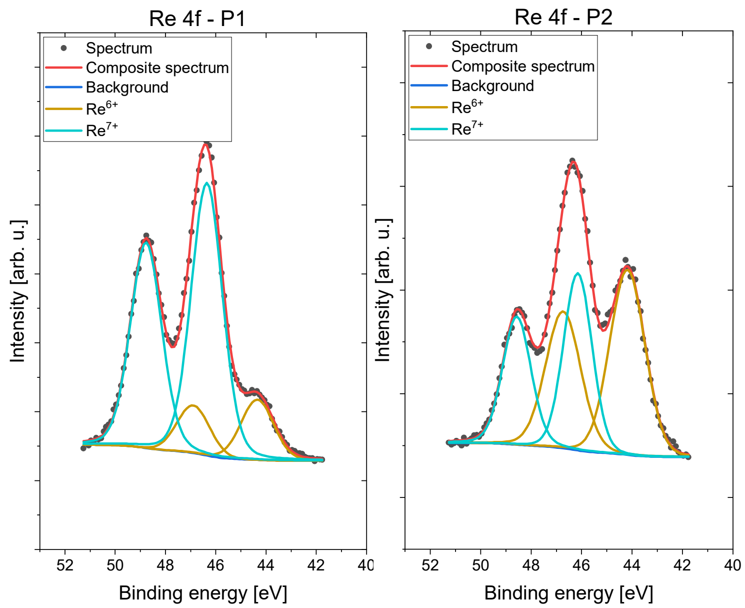

Figure S19. Re 4f spectra of (P1) Catalyst 1, and (P2) Catalyst 2.

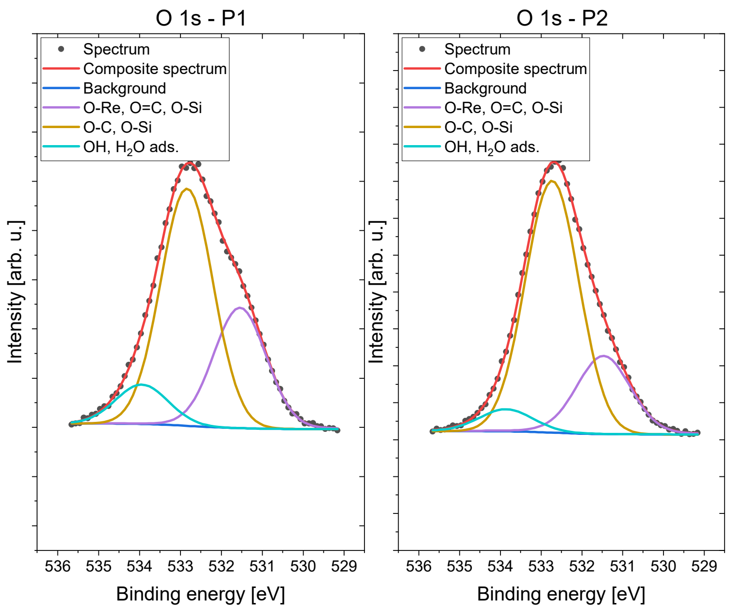

Figure S20.O 1s spectra of (P1) Catalyst 1, and (P2) Catalyst 2.

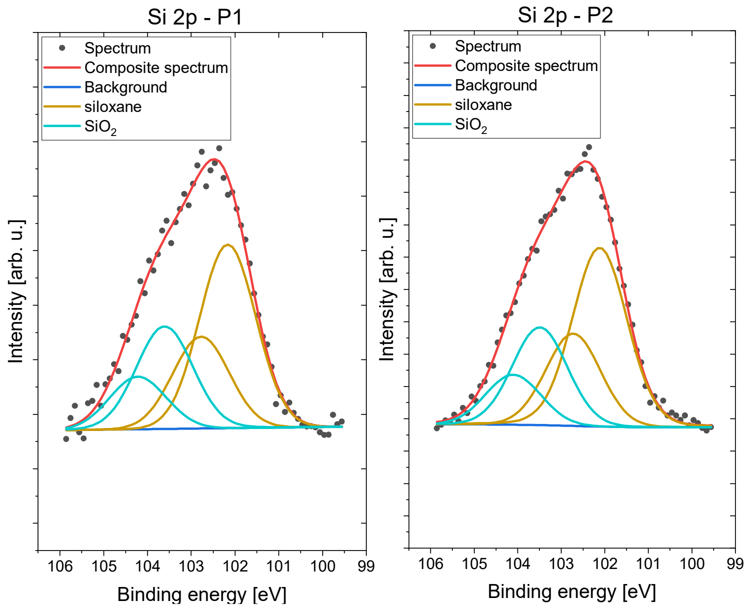

Figure S21. Si 2p spectra of (P1) Catalyst 1, and (P2) Catalyst 2.

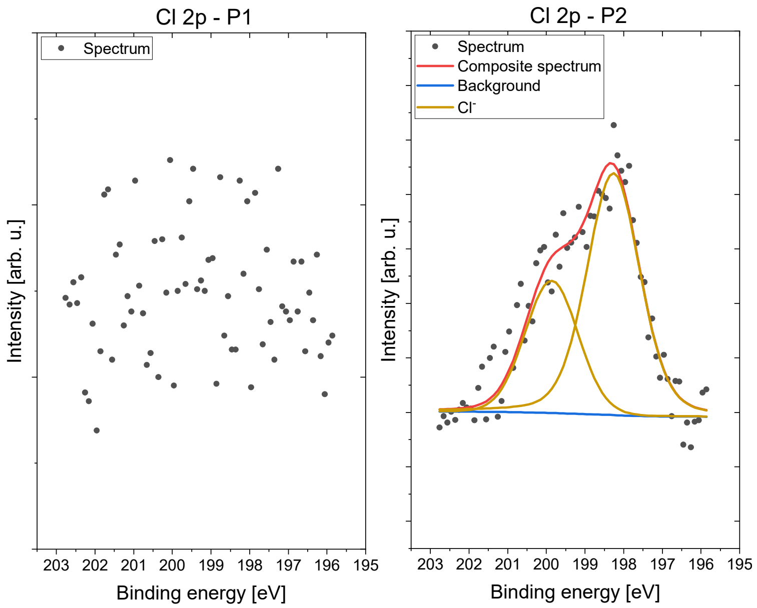

Figure S22. Cl 2p spectra of (P1) Catalyst 1, and (P2) Catalyst 2.
